# Solvent Polarity as a Diagnostic for Vibronic/Vibrational Coherences Distinction in an Efficient TADF‐TTA Switchable Emitter

**DOI:** 10.1002/smsc.70387

**Published:** 2026-08-21

**Authors:** Ajeet K. Singh, M. Sridevi, Dhrubajyoti Paul, Annu Agarwal, Nancy Punia, Rajiv Singh, Inamur Rahaman Laskar

**Affiliations:** ^1^ Department of Chemistry Birla Institute of Technology and Science Pilani Campus Pilani Rajasthan India; ^2^ Photovoltaic Metrological Section Advanced Materials & Device Metrology Division CSIR‐National Physical Laboratory New Delhi India

**Keywords:** intensity borrowings, pump‐probe spectroscopy, thermally activated delayed fluorescence, transition dipole interference, triplet‐triplet annihilation, vibronic/Vibrational coherences

## Abstract

Distinguishing vibrational from vibronic coherences in donor–acceptor (D‐A) emitters remains a central challenge in excited‐state spectroscopy, particularly for thermally activated delayed fluorescence (TADF) systems where temperature‐dependent analysis is often compromised by conformational changes and altered charge‐transfer (CT) populations. Here, we introduce a pump‐probe‐only, solvent‐polarity‐based strategy to resolve this problem using 1,8‐naphthalimide D‐A chromophores. In nonpolar n‐hexane, resonant LE‐CT coupling generates persistent coherent beating, whereas in polar dichloromethane, the CT state is stabilized, the LE‐CT gap widens, and vibronic coherence is quenched while vibrational oscillations remain comparatively robust. This establishes a practical diagnostic for identifying LE‐CT vibronic mixing without recourse to temperature variation or two‐dimensional spectroscopy. Beyond coherence assignment, the same LE‐CT coupling explains intensity borrowing and the appearance of CT absorption even in near‐orthogonal geometries, providing a route to estimate the effective CT transition dipole and to rationalize enhanced optical absorption. Finally, we show unprecedented polarity‐controlled switching between TADF and triplet–triplet annihilation (TTA) in a single deep‐blue emitter, thereby linking coherence physics, LE‐CT electronic structure, and triplet harvesting into a unified framework for designing polarity‐robust optoelectronic materials.

## Graphical abstract

Solvent polarity distinguishes vibronic from vibrational coherence in donor–acceptor emitters: a resonant LE–CT gap in nonpolar media sustains vibronic beating, while polar solvents widen the gap and quench it: a simple, temperature‐free diagnostic for TADF systems.

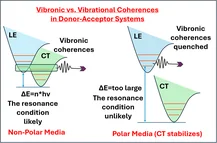

## 1 Introduction

**FIGURE 1:**
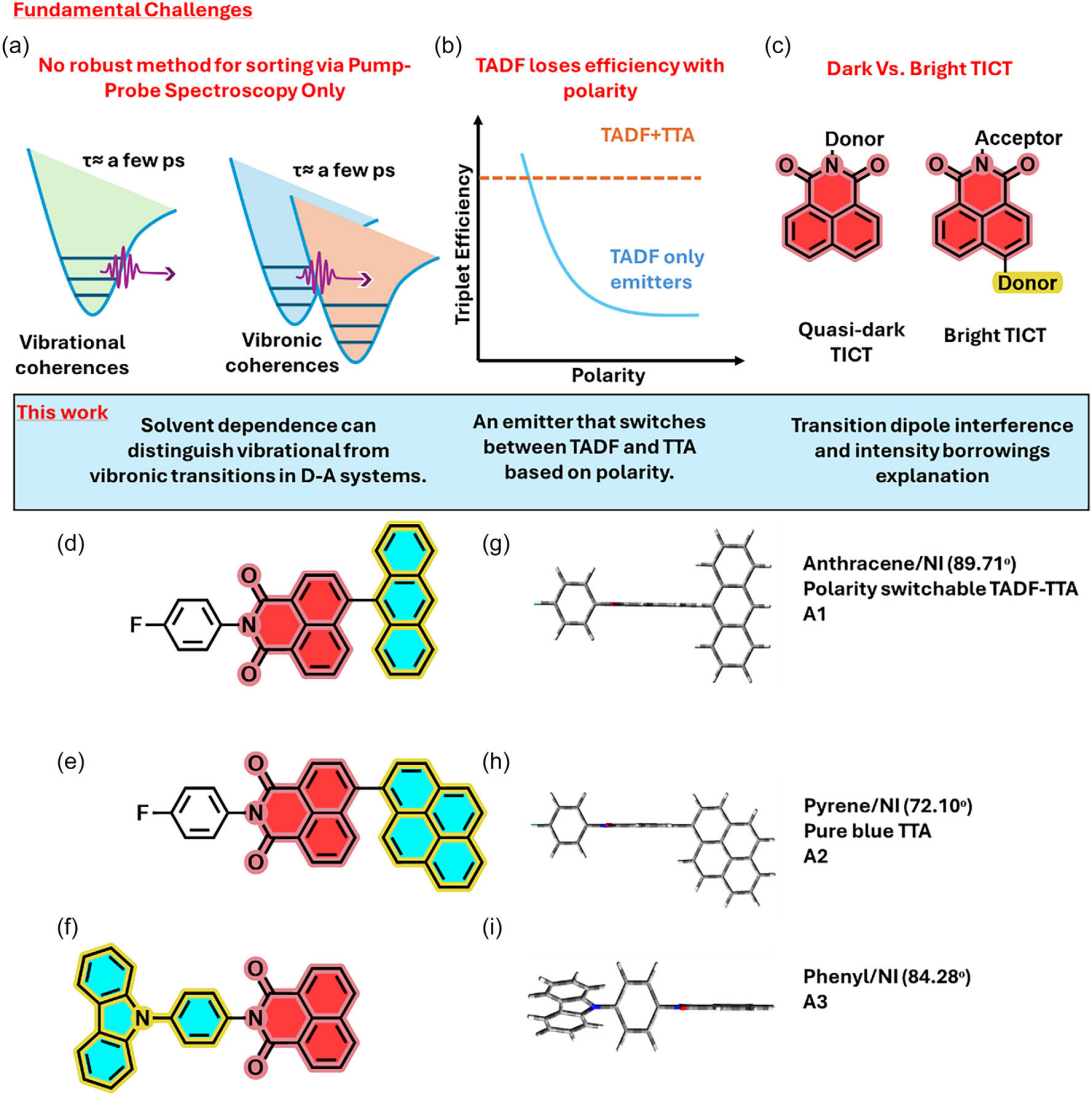
Fundamental challenges in the literature include (a) the distinction between vibronic and vibrational coherences is difficult solely based on pump‐probe experiments due to similar timescales. (b) Traditional TADF suffers from poor triplet efficiency as the polarity of the solvent increases. (c) Dark TICT absorption is observed when D‐A systems adopt an orthogonal geometry. (d–f) Structures of synthesized molecules A1‐A3. (g–i) The optimized molecular structures and the dihedral angles between the donor and acceptor were calculated using quantum‐chemical methods (cam‐B3LYP/6‐311+G(d,p), gas phase; see Computational Studies, Supporting Information).

Coherences play a crucial role in the excited‐state dynamics. They are classified as electronic (between distinct electronic states), vibronic (between electronic states mediated by vibrations), or vibrational (within a single electronic state) [1, 2, 3, 4]. Electronic coherences decay rapidly (<100 fs), while vibrational and vibronic coherences persist for picoseconds with overlapping frequencies and lifetimes, creating a critical bottleneck for assignment using standard pump‐probe spectroscopy alone (Figure 1a) [5, 6, 7]. This distinction typically requires multidimensional techniques such as 2D electronic spectroscopy (2DES) or polarization anisotropy, yet no robust pump‐probe‐only method exists to make it [8, 9, 10, 11].

Temperature dependence is commonly employed, exploiting stronger phase shifts in vibrational coherences arising from thermal population changes rather than the minimal shifts in vibronic superpositions [9]. However, this approach often fails, especially in thermally activated delayed fluorescent (TADF) systems [12]. Typically, in donor–acceptor systems (including TADF), cooling may induce conformational changes that favor a specific geometry and alter/stabilize CT states. This can lead to a shift in energies, which are directly related to vibronic frequencies. Tsuchiya et al. demonstrated that the conformations vary with temperature, with some conformations dominant at specific temperatures [13]. Furthermore, temperature variations can suppress or enhance certain decay channels, thereby altering LE/CT population ratios [14, 15]. Similar solvent‐dependent excited‐state behavior in donor–acceptor exciplex systems has recently been characterized using combined transient‐absorption and DFT approaches [16]. These pitfalls may lead to ambiguous assignments in D‐A systems.

Here, we introduce an unprecedented solvent‐dependent strategy using pump‐probe‐only to distinguish between vibrational and vibronic coherences in D‐A (e.g., A2) and TADF systems (e.g., A1) without temperature dependence. Typically, in D‐A systems, the excited states exhibit both LE and CT character [17, 18]. Vibronic coherences are highly susceptible to the energy gap between the coupled states [19]. Fortunately, CT states are also vulnerable to solvent polarity and stabilize as the dielectric constant increases. Thus, we hypothesize that in D‐A systems, when LE and CT states are vibronically coupled, it is possible to distinguish vibrational and vibronic coherences using only pump‐probe spectroscopy (without 2DES support or temperature dependence) by varying the solvent polarity. In a low‐polarity solvent, the LE‐CT energy gap is expected to be smaller, and the resonance condition is likely to hold; as polarity increases, the LE‐CT gap should also increase. This would likely quench the vibronic coherences. A further diagnostic can be using the dephasing time [20, 21]. Dephasing of a coherence is governed most directly by the spectral density of the environment and the associated reorganization energy, rather than by solvent polarity as such [22], however, in donor–acceptor charge‐transfer systems, polarity and CT‐state reorganization energy are closely correlated; increasing polarity stabilizes the CT state and increases solute‐solvent coupling, making polarity an experimentally convenient, though imperfect, proxy for system‐bath coupling strength in this specific class of system. On this basis, and as a working hypothesis tested directly against the data in Section B, we anticipate that when system‐bath interactions are weak, as expected in nonpolar media, vibronic coherences will tend to dephase more slowly, whereas in higher‐polarity media, where the CT reorganization energy is larger, they will tend to dephase more rapidly. This trend is not universal: solvent relaxation dynamics and specific solute‐solvent interactions can modulate dephasing independently of polarity, and we treat it here as a testable expectation rather than a general rule. This suggests that if the coherences are vibronic in nature, the oscillations are expected to quench and dephase more rapidly as solvent polarity increases. This can be of vital importance for TADF materials and, of course, for other D‐A systems in general (Section 2.2).

Next, TADF emitters enable ~100% exciton utilization via temperature‐sensitive reverse intersystem crossing (RISC), driven by a small singlet‐triplet energy gap (Δ*E*_ST_) [23, 24]. But polarity increases non‐radiative decay and Δ*E*_ST_, restricting their efficiency (Figure 1b) [25]. Triplet–triplet annihilation (TTA) offers an alternative triplet‐harvesting pathway (62.5% theoretical efficiency) via bimolecular diffusion [15, 26], maintaining efficacy in polar media independent of the need for a small Δ*E*_ST_ gap. These two mechanisms are complementary, and if achieved in a single molecule, then it can offer greater flexibility in devices. Despite inherent TTA in organic emitters, weak spin–orbit coupling (SOC < 0.05 cm^−1^) limits triplet populations [3, 27, 28]. SOC can be increased by the carbonyl's nπ*, which is expected to enhance the triplet population [29].

We report the first polarity‐switchable TADF (nonpolar) → TTA (polar) mechanism in deep‐blue NI emitter A1. This dynamic triplet‐harvesting approach overcomes the polarity limitations of traditional CT‐based TADF. This can offer versatile, polarity‐robust OLED performance. Moreover, such dynamic phenomena can also enhance overall device performance. When TADF is restricted by energetic or similar constraints, the triplet population can be recovered via TTA [30]. Thus, TADF–TTA probes would be ideal for next‐generation OLED devices (Section 2.3).

This vibronic coupling insight resolves a peculiar observation in the 1,8‐naphthalimide (NI) derivatives literature (Figure 1c): at times, TICT geometry yields a dark absorption, and authors attribute it to orthogonality [31, 32, 33, 34, 35, 36, 37, 38]. At other times, a clear CT band is seen, even at orthogonal geometries [39]. No clear explanation is available in the literature; resolving these ambiguities is fundamental to understanding TICT photophysics, which could lead to better materials for optoelectronics (TICT geometry is an important aspect of CT‐based TADF molecules) and bioimaging. Here, we show that couplings are important, as they can significantly alter photophysics. This mixing leads to the phenomenon of intensity borrowing, that is, exchange of intensity between coupled states [40]. We explain how TICT absorption goes dark and when it appears bright (Section 2.1).

All these gaps are interrelated, and 1,8‐naphthalimide (NI) derivatives are perfectly suited to address them (Figure 1d–f). These derivatives are classic D‐A systems and have been previously reported to exhibit strong vibronic coupling between LE (NI ring‐localized) and CT states, thereby allowing us to address the first gap via polarity sensitivity [29, 41]. Then, the two carbonyl groups should provide reasonable SOC and, along with orthogonality, enable polarity‐dependent TADF‐TTA switching in a single molecule. Lastly, they offer easy access to TICT geometry (A1 and A3) via opposite‐end substitution to fill the third gap. A1 and A2 are novel molecules, whereas A3 has been previously reported [42].

In donor–acceptor systems, including TADF emitters, LE and CT states often lie close in energy, and therefore distinguishing whether the observed coherences are vibrational or arise from genuine vibronic coupling is of fundamental importance. This distinction is crucial because vibronic coupling implies direct electronic communication between LE and CT manifolds, whereas purely vibrational coherence does not. Once LE and CT states are vibronically mixed, their photophysics cannot be understood independently, as such mixing can alter resonance conditions, coherence lifetimes, transition‐dipole interference, and the extent of intensity borrowing from bright LE states into weakly allowed CT states.

This has consequences far beyond coherence assignments alone. In TICT chromophores, CT absorption is often assumed to become dark simply because the donor and acceptor approach orthogonality, yet the present framework suggests that LE‐CT coupling can strongly modulate whether a CT/TICT transition appears bright, weak, or quasi‐dark. Thus, identifying vibronic coupling provides mechanistic insight into the origin of CT oscillator strength and offers a practical, qualitative route to infer the effective orientation and contribution of the CT transition dipole relative to the fixed LE transition dipole of the acceptor core. Such knowledge is essential for understanding how to enhance optical absorption in weakly allowed CT systems and for rationalizing why structurally similar D‐A derivatives can show markedly different low‐energy absorption behavior.

At the same time, the LE‐CT energetic landscape also governs triplet harvesting in these materials. Because solvent polarity tunes CT stabilization, it can simultaneously control vibronic resonance, dephasing, Δ*E*_ST_, and the balance between TADF and TTA pathways. Therefore, establishing whether LE and CT states are vibronically coupled not only resolves a fundamental spectroscopic problem but also provides a design principle for developing D‐A emitters with stronger absorption, improved triplet utilization, and polarity‐dependent switching between complementary delayed‐emission channels. In this sense, the distinction between vibrational and vibronic coherence unifies the three central questions addressed here: coherence assignment, the brightness or darkness of CT absorption, and the realization of a polarity‐switchable TADF–TTA emitter within a single molecular platform.

An older version of this article was submitted to ChemRxiv (10.26434/chemrxiv.15001110/v2).

## 2 Results and Discussion

### 2.1 Section A

#### 2.1.1 UV/Vis Spectroscopy: Intensity Borrowings and Transition Dipole Interference

**FIGURE 2:**
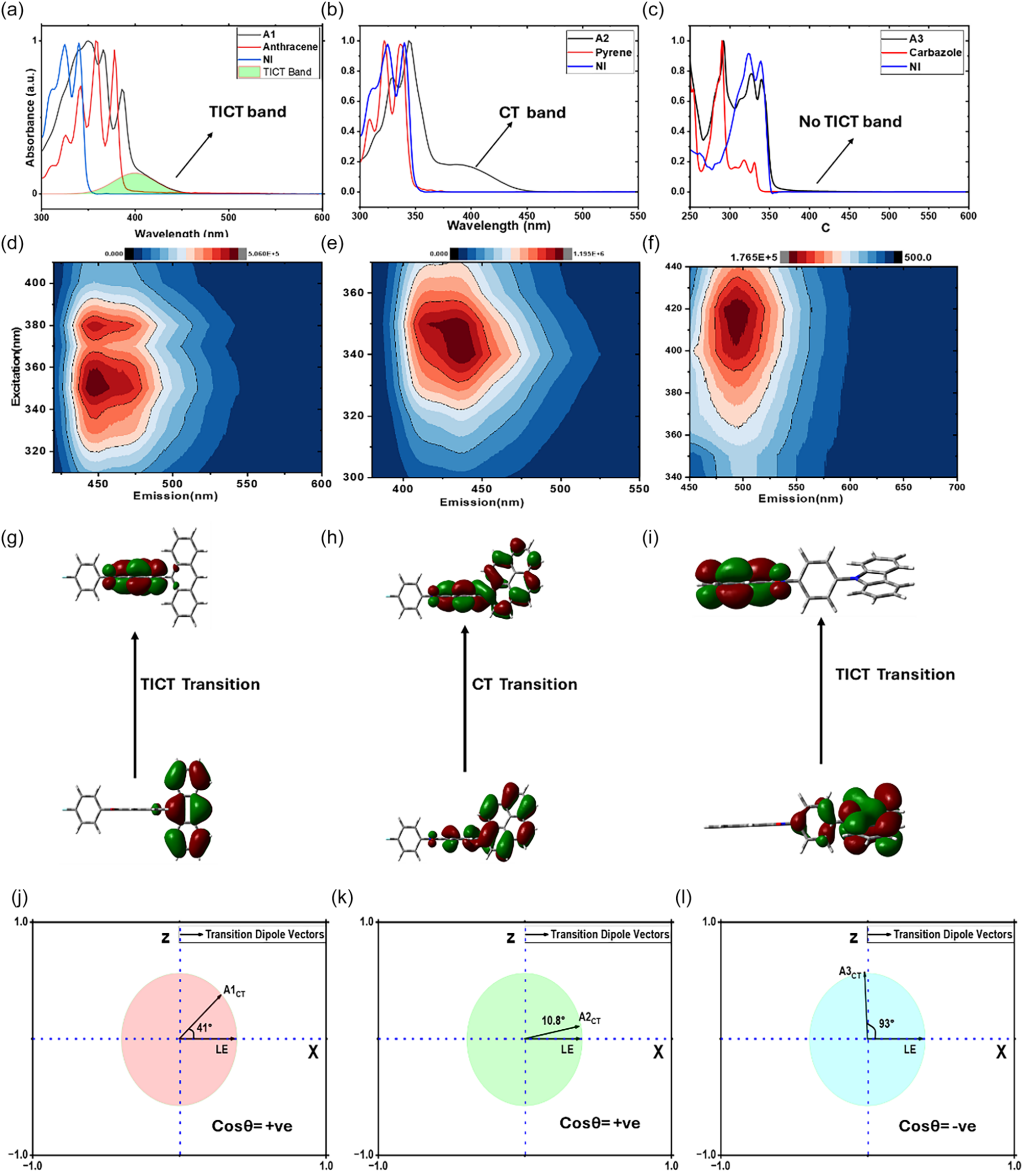
Absorption spectra of the A1 (a), A2 (b), and A3 (c) molecules showing distinct CT absorption. (d–f) Excitation‐dependent emission contours of A1–A3, with A3 showing a clear band above 400 nm (color shows intensity in a.u.). (g–i) The electron–hole pair is involved in the TICT absorption of the molecules. (j–l) Transition dipole vectors of the CT and LE transitions of each molecule, showing the angle between TDMs. A1 and A2 are experimentally determined, while A3 is computationally calculated.

Absorption spectroscopy of the synthesized molecules A1–A3, along with their respective donors (anthracene, pyrene, and carbazole, respectively) and the NI acceptor, was carried out in n‐hexane (Figure 2a–c). A1 and A2 exhibited an isolated, small, broad, lower‐energy band in the 400–450 nm region, attributed to a TICT state for A1 and a CT state for A2, respectively. The dihedral angle in A2 is ~72°; hence, we will not use the term TICT for this particular case. Additionally, a high‐energy band that matched either the absorptions localized on the NI ring or the donors. The high‐energy band (350–400 nm) in A1 matched the anthracene local transitions, with slight redshifts and additional contributions from the NI local transitions in the 300–350 nm region. Thus, *S*_2_ is assigned to anthracene‐localized transitions, while S_3_ transitions are NI‐localized. A2 had contributions from both pyrene and the NI ring in the 300–375 nm region. These bands were assigned to local transitions on the donor or the NI ring, depending on whether the peak matched the donor or the acceptor. The slight redshifts in the LE transitions, compared to those of the donor and acceptor units in both, are attributed to the compounds’ extended conjugation. In contrast, A3 showed a strong absorption band in 330–360 nm, which matched the NI rings’ absorption and was assigned to LE transitions on the NI ring. Notably, no absorption was seen beyond 375 nm in this case.

To confirm if there is any absorption beyond 375 nm in A3 and to get suitable excitation for emission, we collected excitation versus emission contours for each molecule (Figure 2d–f). Both A1 and A2 showed suitable excitation at 350 nm. Interestingly, A3 unambiguously exhibited a clear emission when excited above 400 nm. This is consistent with quasi‐dark behavior (i.e., absorption is not strictly zero but is invisible in UV/Vis spectroscopy).

Computational calculations were performed (details are provided in the methodology section of the ESI). The time‐dependent Density Functional Theory (TDDFT) calculations of A1 found three low‐energy transitions within 0.49 eV. *S*_1_ was found to be an LE transition with transitions localized on the anthracene ring (*f* = 0.2301), and S_2_ is a TICT (*f* = 0.0002, Table S3) with charge transfer from anthracene → NI (Figure 2g), and S_3_ was also LE with transitions localized on the NI ring (*f* = 0.3284). Experimentally, *S*_1_ is a TICT state, and *S*_2_ is an anthracene‐localized state. A3 calculations revealed that the S_1_ transition was NI localized (0.3405), while the *S*_2_ transition had TICT character (phenyl carbazole → NI with negligible oscillator strength). Here, too, the experiments show that the quasi‐dark TICT state is *S*_1_, while the NI‐localized transitions are *S*_2_. This energy mismatch is attributed to the shortcomings of standard TD‐DFT in the vibronic coupling regime. In general, when couplings are involved, TDDFT energy ordering becomes unreliable, and this is evident in our calculations, too, since standard TDDFT does not account for couplings [43, 44]. In A2, the energy ordering matches experiments. The *S*_1_ transition of A2 involves several orbitals with *f* = 0.6585; we calculated the natural transition orbitals (NTOs). NTOs show a major CT character (pyrene →NI) in the first singlet transition of A2. Figure 2g–i shows the orbitals involved in the CT transition of each compound.

The UV/Vis spectra of A1 and A3 reveal a clear difference in TICT absorption behavior depending on whether the donor is attached to the naphthalene ring or to the imide. A recurring trend in the literature is that donor–acceptor molecules bearing donors directly attached to the imide position often exhibit very weak or absent CT absorption, which is commonly attributed to near‐orthogonal donor–acceptor geometries [31, 32, 33, 35, 36, 37, 38, 41, 45, 46, 47, 48, 49, 50, 51, 52, 53]. In the present series, however, A1 exhibits a distinct TICT‐related band despite a highly twisted geometry, whereas A3 shows only a weak, quasi‐dark low‐energy feature. This indicates that orthogonality alone is insufficient to explain the observed trend. A question may arise: are the two donors different? However, changing the donor in A3 analogs doesn’t alter the absorption behavior (absorption beyond 370 nm remains undetected in UV/Vis spectroscopy). In fact, it remains consistent across donors, as we note in the literature [36].

A useful qualitative framework is provided by the effective transition‐dipole expression, *μ*_eff_ = *μ*_S1_ + α cos*θ* *μ*_S2_, where the observed intensity depends on the magnitudes and relative orientations of the CT and LE transition dipole moments [54]. Within this framework, the transient/coherence data are informative (vide infra). The phases of coherent oscillations are directly related to the TDM of the coupled states [55]. Experimentally, *S*_1_ and *S*_2_ oscillatory components are nearly phase‐aligned in A1 and A2, with phase differences of 41° and 10.8°, respectively (Figure 2j–l). This means even if the first term (*μ*_S1_) is negligible due to orthogonality, the transition gains oscillator strength from the second term (+αμ_S2_), thus explaining a clear band in A1 despite orthogonality. This is consistent with favorable LE–CT mixing and partial intensity borrowing from the bright LE state.

For A3, the DFT calculations were performed, and the calculated transition‐dipole vectors for the relevant low‐energy states provide additional qualitative support. The S_1_ transition dipole is (1.8582, 0.0000, −0.0001), while the corresponding S_2_ transition dipole is (−0.0009, −0.0030, 0.0153), yielding an inter‐vector angle of approximately 93°. This places the two transition dipoles just beyond orthogonality and suggests a slightly anti‐aligned arrangement in the simplified vector picture, consistent with a weakly destructive contribution to the net intensity. In D‐A systems, the CT transition dipole is generally oriented from donor to acceptor, while the LE transition dipole of the NI unit has been reported previously [41, 53, 56, 57, 58]. Our DFT results of A3 are consistent with this. At the same time, because TDDFT is known to be less reliable in strongly coupled vibronic regimes, especially for near‐degenerate LE/CT manifolds, this result should be interpreted with caution and primarily as qualitative support rather than a quantitative proof. Nevertheless, the calculated orientation is in reasonable agreement with the experimentally observed weak low‐energy absorption of A3, as well as with the literature assignment of the NI‐localized LE transition and the donor‐to‐acceptor CT direction.

Taken together, these results suggest that the observed TICT brightness or darkness may extend across the present derivative series, with the site of donor attachment strongly influencing the net absorption intensity. Since the LE transition dipole of the NI unit is essentially fixed, whereas the CT transition dipole is generally oriented from donor to acceptor, the relative orientation between these two contributions can vary from one derivative to another. This may provide a plausible basis for the strong TICT absorption observed in the A1 analogs and the much weaker, quasi‐dark response in the A3 analogs. Accordingly, the weak or absent TICT absorption in D‐A systems should not be attributed solely to orthogonality; rather, LE–CT coupling and possible intensity borrowing may also play an important role in determining whether a TICT state appears bright or dark.

Next, we explore the vibronic couplings in these derivatives. Henceforth, experiments will be conducted only on A1 and A2.

### 2.2 Section B

#### 2.2.1 Ultrafast Transient Absorption Spectroscopy

**FIGURE 3:**
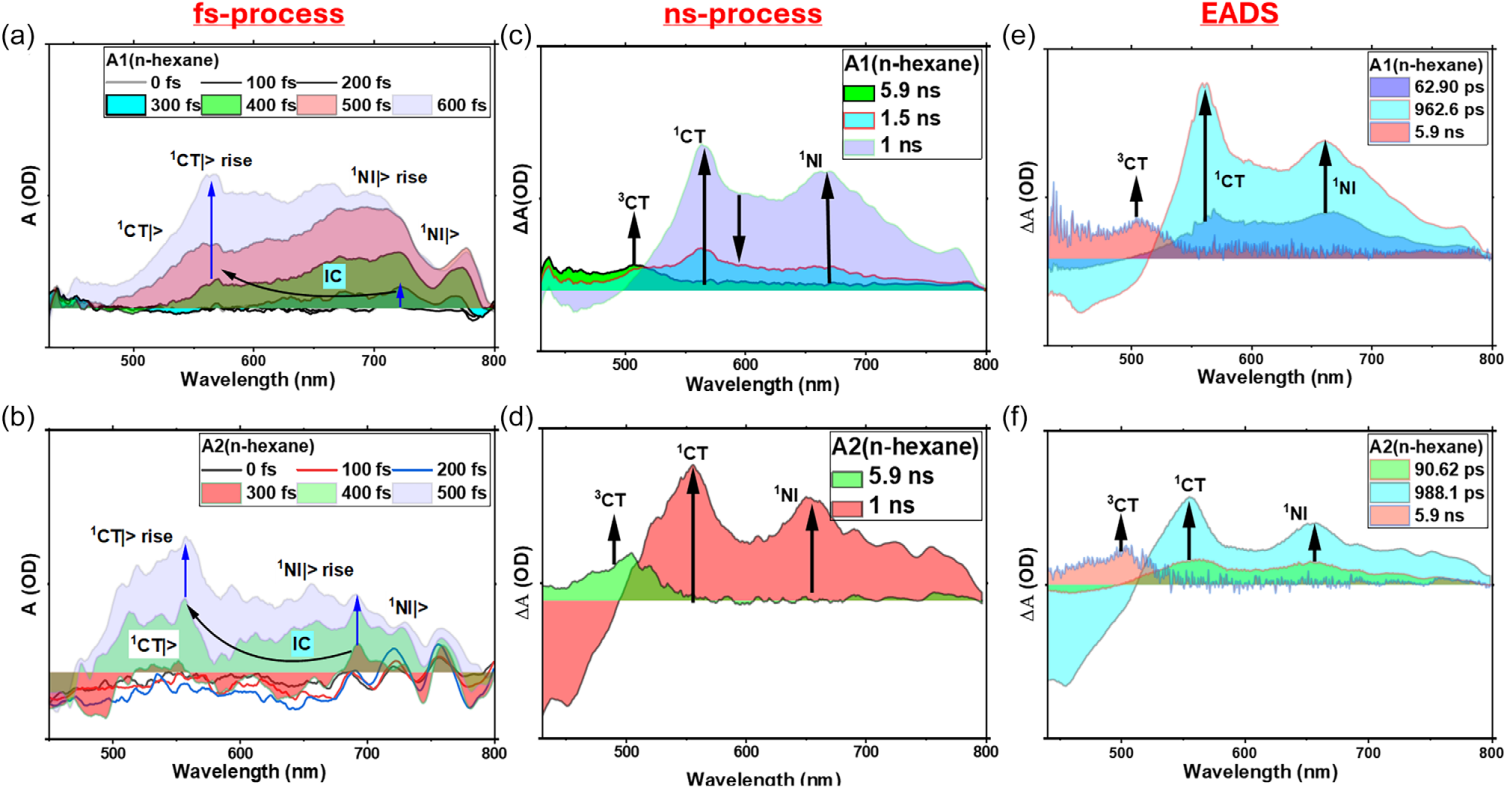
(a–d) Raw transients of A1 and A2 in n‐hexane at various timescales. (c,d) EADS spectra obtained by global analysis show three prominent components.

Now, we explore the ultrafast dynamics of excited states. A 35‐femtosecond pump‐probe spectroscopy was performed in n‐hexane over probe delays up to 5.9 ns. Raw transient plots at various timescales are given in Figure 3a–d. We present the A1 results, while the A2 results are equivalent. The excitation wavelength was 350 nm to ensure that the excited‐LE state (NI‐localized) was probed, along with its relaxation to the low‐lying singlet and triplet manifolds. At the earliest time scale (~200 fs), no transient signal was observed, which was likely due to internal vibrational relaxation (IVR) to the adiabatic minimum of ^1^NI. Then, the ^1^NI population builds up (300 fs), leading to an excited‐state absorption (ESA) band in the 600–800 nm region, consistent with literature on similar NI derivatives in nonpolar solvents [59]. The ^1^NI population undergoes internal conversion (IC) to the CT state after 400 fs, as indicated in Figure 3a,b. It is worth noting that the 500–600 and 600–800 nm bands arise from different electronic states, as their ESA rates differ. In fact, at 500 fs probe delay, the NI band (600–800 nm) has a higher population, but at 600 fs, the 500–600 nm band dominates. This provides strong evidence that these two bands arise from distinct states, and we assign the 500–600 nm band to the CT state, while the 600–800 nm band is due to ^1^NI, consistent with previous reports on similar derivatives [59]. Then, ESA from these two states builds up to 1 ns and decreases afterward. At ~1.5 ns, a completely new band appears in the 450–550 nm region. Surprisingly, this band is present up to 5.9 ns. To understand the origin of this band, we performed time‐resolved spectroscopy to get the steady‐state lifetime of the CT states (Figure S11). A1 has a 7.1 ns steady‐state lifetime, but A2 shows a shorter 2.27 ns timescale. Thus, the band appearing beyond 1.5 ns and living up to 5.9 ns must be associated with the triplet manifold. The steady‐state lifetime of A1 is longer, but its delayed ESA band position and timescale are similar to those of A2, allowing us to safely assign this delayed component to ^3^CT in both cases. For clarity, throughout the remainder of the manuscript, we use ^3^CT to denote a triplet state with substantial charge‐transfer character, analogous to the CT singlet discussed above, and ^3^LE to denote a triplet localized on a single fragment (donor or NI acceptor). As established below (Table 1, Figures S29, and S30), the T_1_ states relevant to the delayed emission and TADF/TTA behavior of both A1 and A2 are best described as predominantly ^3^CT in character, and we use this label consistently in the sections that follow. Thus, in these molecules, intersystem crossing (ISC) is an important channel, driven by the NI ring, which induces strong SOC between the singlet and triplet manifolds (Table S8).

**TABLE 1:** Singlet‐triplet energy gap (Δ*E*_ST_) of the molecules, calculated using the onsets of Figures 8e,f and S29.

Compound	***S*** **_1_** **, eV**	***T*** **_1_** **, eV**	Δ*E*_ST_, eV
**A1 (n‐hexane)**	2.95	2.87	0.08
**A1 (DCM)**	2.59	1.93	0.66
**A1 (DMSO)**	2.41	1.88	0.53
**A2 (n‐hexane)**	3.19	2.67	0.52

*Note:* These values are derived entirely from experimental steady‐state and time‐gated emission onsets, not from computation.

Next, we calculated evolution‐associated difference spectra (EADS) using a global fitting method, which are presented in Figure 3e,f. EADS spectra show three prominent components. First, a component in which the ^1^NI‐^1^CT population is approximately equal, with a lifetime of 62.90 ps. Then, another component shows ^1^CT with higher intensity than ^1^NI, with a timescale of 962.6 ps, and, lastly, the completely new long‐lived band with an infinite lifetime (up to which data were collected, i.e., 5.9 ns). Notice that in the long component, ESA beyond 550 nm is visible only for A1, while for A2, it is negligible. This is because the steady‐state population persists beyond 5.9 ns in A1, whereas the steady‐state decay in A2 is much faster. The assignment of bands is similar to that in raw transient analysis.

#### 2.2.2 Signature of RISC in DCM

**FIGURE 4:**
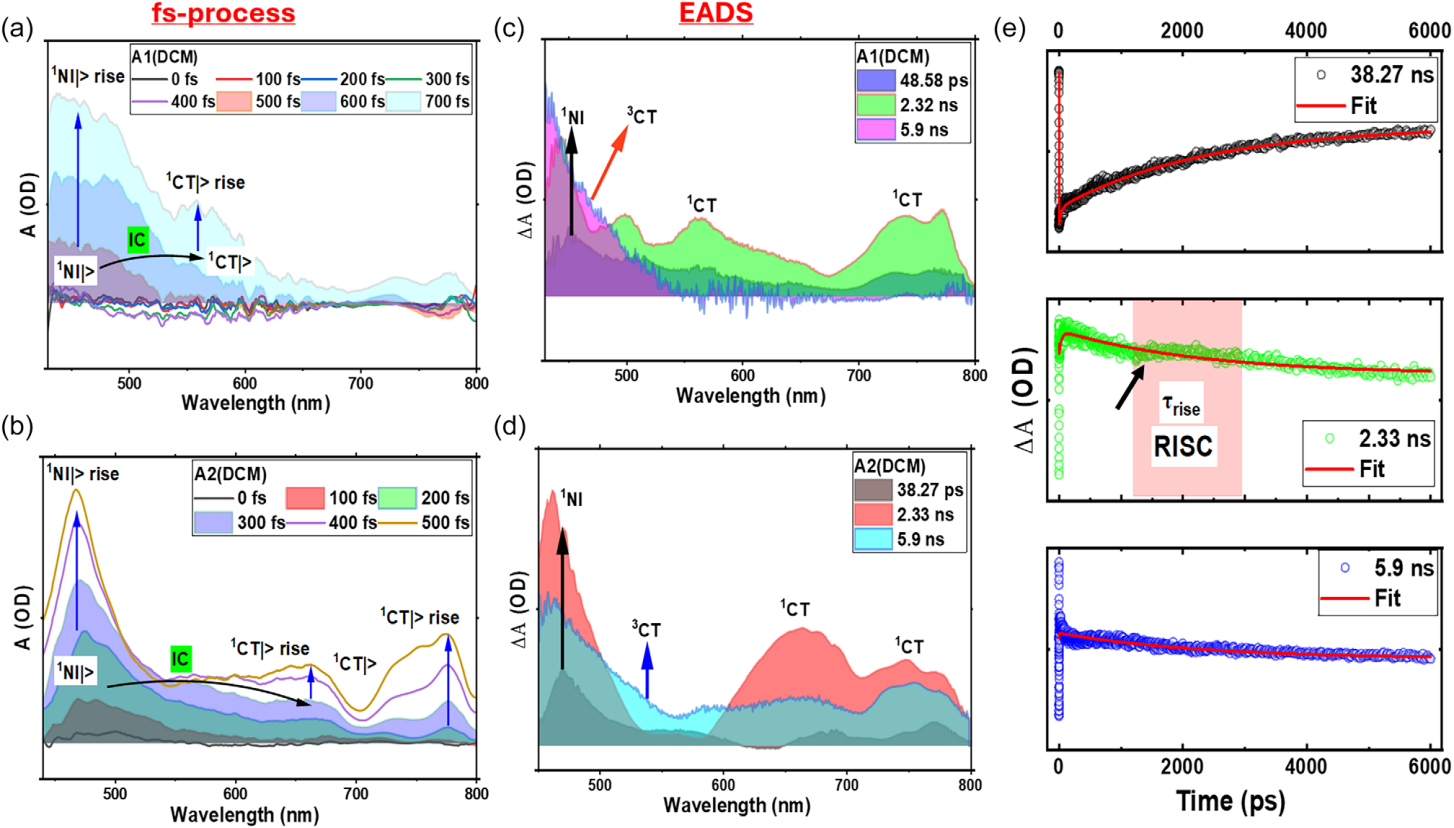
(a,b) Raw transients of A1 and A2 in DCM at various timescales. (c,d) EADS spectra obtained by global analysis show three prominent components. (e) Global kinetics of A2 in DCM. 2.33 ns component shows a rise in population in the shaded region, indicating RISC.

In dichloromethane (DCM) solvent, IVR is much slower in A1 (400 fs) while much faster in A2 (100 fs), as shown in Figure 4a,b. Then, a ^1^NI peak appears in the 450–550 nm region. Then, broad ESA peaks appear in both cases: one in the 550–650 nm region for A1 at 600 fs, and one in the 700–800 nm region for A2 at 200 fs. This is attributed to IC from ^1^NI to the CT state of both molecules. Notably, in DCM, the ESA band of the ^1^CT state is lower than in ^1^NI. The peak assignments are consistent with the literature [59]. While the dynamics in both (A1 and A2) are similar, the ESA band positions and timescale vary drastically.

Next, we carried out EADS analysis (Figure 4c,d). The EADS shows three prominent components. The earliest component, with 48/38 ps for A1/A2, represents the early population in both NI and CT states. Then, these peaks rise in energy, and the component has a lifetime of ~2.3 ns. The longest‐living component is the most interesting one. The steady‐state lifetime of both A1 and A2 for NI and CT peaks is below 4.64 ns. In A1, the longest‐living component is present in the 450–550 nm region, and no contribution from the CT band is observed (500–800 nm). In contrast, A1 clearly shows ESA in the CT region along with the 450–550 nm ESA peak. The ESA peak in the 450–550 nm region in both cases can be safely assigned to ^3^CT. But the ESA in the ^1^CT region in A2 persists beyond its steady‐state lifetime and remains strong. We assign this to delayed fluorescence (DF), via RISC in this molecule. This also, to some extent, rationalizes the unusually longer steady‐state lifetime of CT emission in DCM as compared to n‐hexane. It is generally expected that the timescale of molecules in polar media should be less than that of the polar dielectrics due to enhanced non‐radiative decays [60, 61]. Thus, we believe that some contribution from DF is present in the steady‐state lifetime of the A2 molecule, leading to its enhanced lifetime in DCM. Now, to be absolutely confident about the RISC process, we look into the decay kinetics of the 2.33 ns band. This band is selected because it represents the singlet dynamics. If the decay is gradual, the long‐lived ESA in this region is most likely due to the residual population of these states, since the steady‐state lifetime is the average lifetime and does not reflect the time when the excited‐state population is zero. In contrast, if the population rises after a certain time, the long‐lived ESA bands can be safely assigned to DF due to RISC. Figure 4e clearly shows a *τ*_rise_ period after ~1.3 ns (indicated by the red‐shaded region). This supports assigning the ESA from CT states at longer times to DF.

With all the peaks thoroughly assigned and ultrafast processes studied, we now turn to the molecules’ coherent dynamics.

#### 2.2.3 Vibronic Versus Vibrational Coherences

**FIGURE 5:**
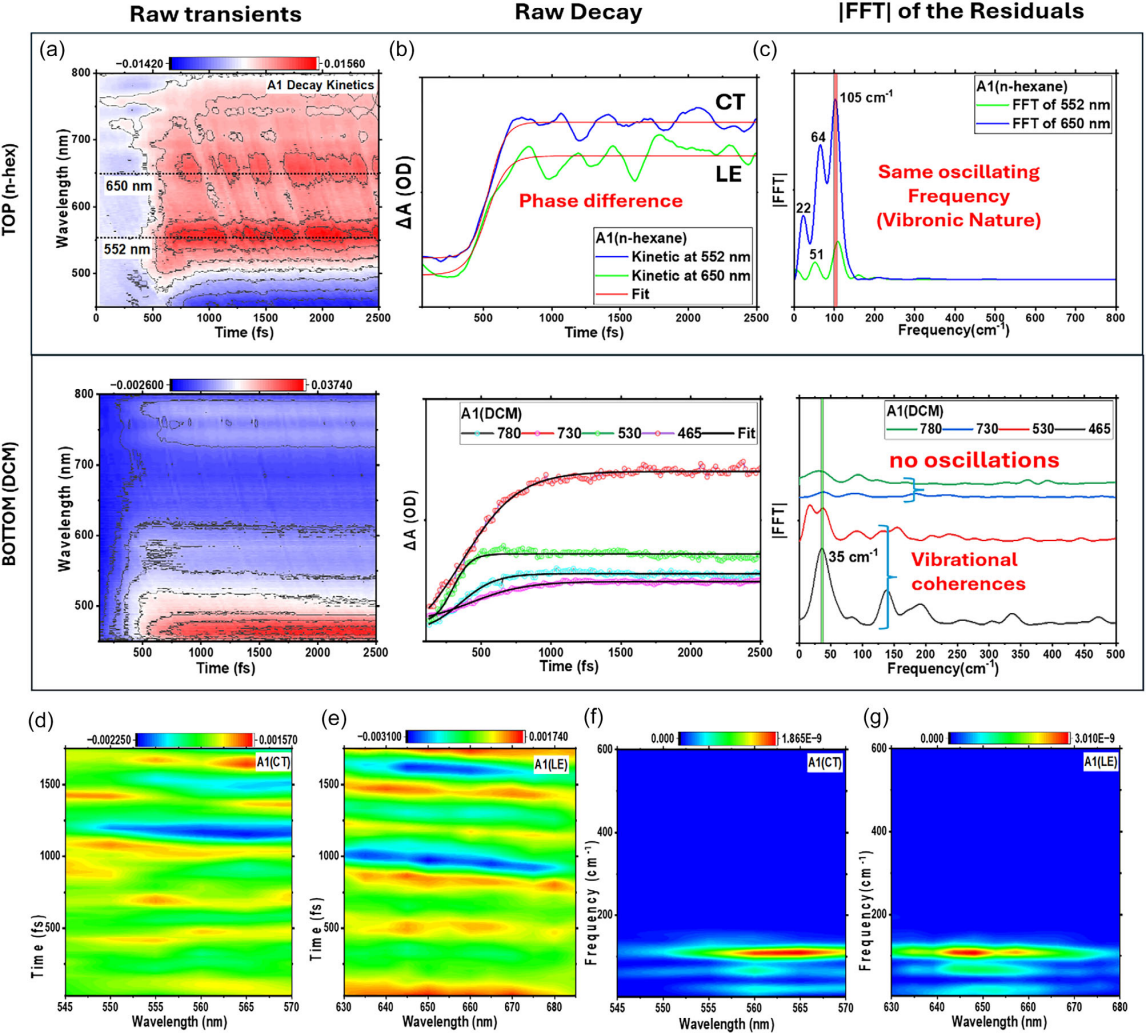
(a) Decay contours of the A1 molecule in the first 2 ps, with dotted line showing the probed wavelength used to extract oscillations (**TOP** shows n‐hexane contours), while not obvious in **BOTTOM** (DCM contours). (b) Decay kinetics showing clear modulations superimposed in n‐hexane (smoothed to 12 data points), but no such oscillations are observed in DCM. (c, **top**) The FFT power spectrum of the residuals obtained by subtracting the fits from the decay kinetics shows a dominant component in both the CT and LE bands, assigned to vibronic coherences (**TOP**). (**C, bottom**) The CT band shows no oscillatory features, but the LE band shows coherence and is assigned a vibrational nature. (d,e) Residual contours of the decay kinetics show consistent oscillations in the entire band in n‐hexane. (f,g) FFT power contours of the residuals show a dominant oscillatory frequency in the entire band in n‐hexane. (*All the color bars in contour plots show* Δ*A in OD.*).

Now, we explore the coherent dynamics of the molecules and distinguish between vibrational and vibronic coherences in the systems. For this purpose, we shortened the probe delay up to 90 ps and increased the number of data points. The decay kinetics were probed at the earliest timescale (the first 2–3 ps), and the contour plots are shown in Figure 5a, top. Again, we explain the A1 results; A2 is equivalent and is shown in Figure S15. Raw contour plots unambiguously reveal that the ESA oscillates over the first 2000 fs in both cases. In general, the populations follow a simple mono‐ or multiexponential pattern, either decaying or rising. In some cases, additional population growth can occur from other states, or leaks to other states may occur. However, the population does not oscillate unless coherence is involved, that is, two states are in superposition (coherence) [62]. These oscillations result from the interference of the coupled states, manifesting as superimposed oscillations on the decay kinetics. This is consistent with coherence phenomena [63, 64]. Notably, these oscillations were probed in the ESA spectral window, indicating an excited‐state origin rather than ground‐state coherences. To further analyze these oscillations, we probed both LE‐CT (henceforth, ^1^NI will be represented as LE while ^1^CT will be simply called CT) ESA peaks at specific wavelengths. This is shown by dashed lines in the plots: 655 and 552 nm for A1, and 650 and 550 nm for A2. These kinetics were extracted and fitted with multiexponential functions (Figure 5b). The kinetics show clearly that the decay signals modulate around the fitted curve. To confirm the oscillations and determine the modulation frequency, the fits were subtracted from the decay kinetics to obtain the residuals (Figures S16 and S18). These residuals were further subjected to a Fast Fourier Transformation (FFT, Figure 5c). The FFT power spectrum revealed a dominant component, consistent with the presence of true oscillations. The oscillatory frequency for both LE and CT is the same (105 cm^−1^ for A1 and 110 cm^−1^ for A2).

To probe the entire LE and CT bands, the decays were extracted and fitted. The residual contours are given in Figure 5d,e. The residuals unambiguously show robust coherent oscillations in the entire band. When the residuals were subjected to FFT, the entire band showed a dominant oscillatory feature at the same frequency (Figure 5f,g), matching the oscillatory frequency and pattern of the single probed wavelength. Frequency‐matching between the LE and CT bands is, on its own, necessary but not sufficient evidence for vibronic coupling: the same low‐frequency oscillation could in principle, also arise from (a) a vibrational wavepacket launched impulsively on a single excited‐state surface that projects into both spectral windows simply because the LE and CT ESA bands overlap, or (b) nuclear wavepacket motion on coupled excited‐state potential‐energy surfaces. We can exclude interpretation (a) on two grounds. First, a spectrally overlapping single‐mode wavepacket of this kind would not be expected to depend on the LE–CT electronic energy gap, whereas the oscillatory amplitude tracks the solvent‐dependent LE–CT resonance condition closely (*n* ≈ 9 in n‐hexane, moving out of resonance in DCM, as discussed below), which is the expected signature of coupling between the two electronic characters rather than of a spectator vibrational mode. Second, the LE and CT oscillations are not perfectly in phase: the sine‐fit analysis (Figure 7; see Phase relationship of the LE–CT coherences, below) yields a small but finite phase offset (~41° for A1 and ~10.8° for A2) rather than the near‐zero offset expected if both bands were simply reporting an identical, impulsively driven wavepacket. This use of relative phase in a frequency‐integrated pump‐probe signal to distinguish coupled/coherent dynamics from simple vibrational wavepacket motion follows the framework of Yuen‐Zhou et al. [7], which was later applied in a practical experimental test by Johnson et al. [65] Interpretation (b), nuclear motion on coupled excited‐state surfaces, is not in fact distinct from vibronic mixing as we use the term here: such coupled‐surface motion is precisely what manifests spectroscopically as a shared oscillatory frequency with a finite inter‐band phase relationship. These distinct LE and CT states would typically exhibit unique vibrational structures and distinct oscillatory frequencies in their ESA bands. The shared frequency observed here, together with the phase and solvent‐polarity evidence discussed above, therefore supports vibronic mixing between CT and NI‐localized (LE) transitions rather than proving it outright; we return to the converging evidence of solvent dependence and phase in the following two subsections.

Now we present the coherent dynamics in polar media (DCM) at 350 nm excitation in the earliest timescale (first 2–3 ps). The contours shown in Figure 5a bottom reveal several ESA features. Notably, no oscillatory pattern was observed in these contours. To confirm whether any oscillatory component is present in any of the ESAs, we extracted the decay at the prominent features (465, 530, 730, and 780 nm for A1, and 480, 510, and 750 nm for A2). The extracted kinetics were fit (Figure 5b‐bottom), and residuals were extracted (Figure S19). Residuals show distinct features in LE and CT states. CT shows no oscillatory features, whereas LE shows weak oscillations that persist beyond 2000 fs. On applying the FFT to the residuals, the redshifted bands exhibited no oscillatory features, while the low‐frequency oscillations were present in higher‐energy bands (Figure 5c‐bottom). To understand the origin of these coherences, we turn to system‐bath interactions and dephasing rates. As discussed in the Introduction, solvent polarity is not itself the physical driver of dephasing, but it correlates with the CT‐state reorganization energy in donor–acceptor systems, such that more polar solvents are expected to interact more strongly with the system, leading to faster dephasing rates [22, 66]. Thus, we expect the vibronic coherences in DCM to have a shorter lifetime than in n‐hexane (~1750 fs), if they were vibronic in character. However, these 35 cm^−1^ oscillations in DCM persisted for more than 2000 fs, well beyond the expected vibronic dephasing timescale, which is more consistent with a vibrational rather than a vibronic origin (Figure S19a,b). Thus, the oscillations were assigned to vibrational coherences on this basis, while we note that this assignment rests on the expected polarity‐dephasing trend discussed above rather than on a direct, independent measurement of the reorganization energy in DCM.

**FIGURE 6:**
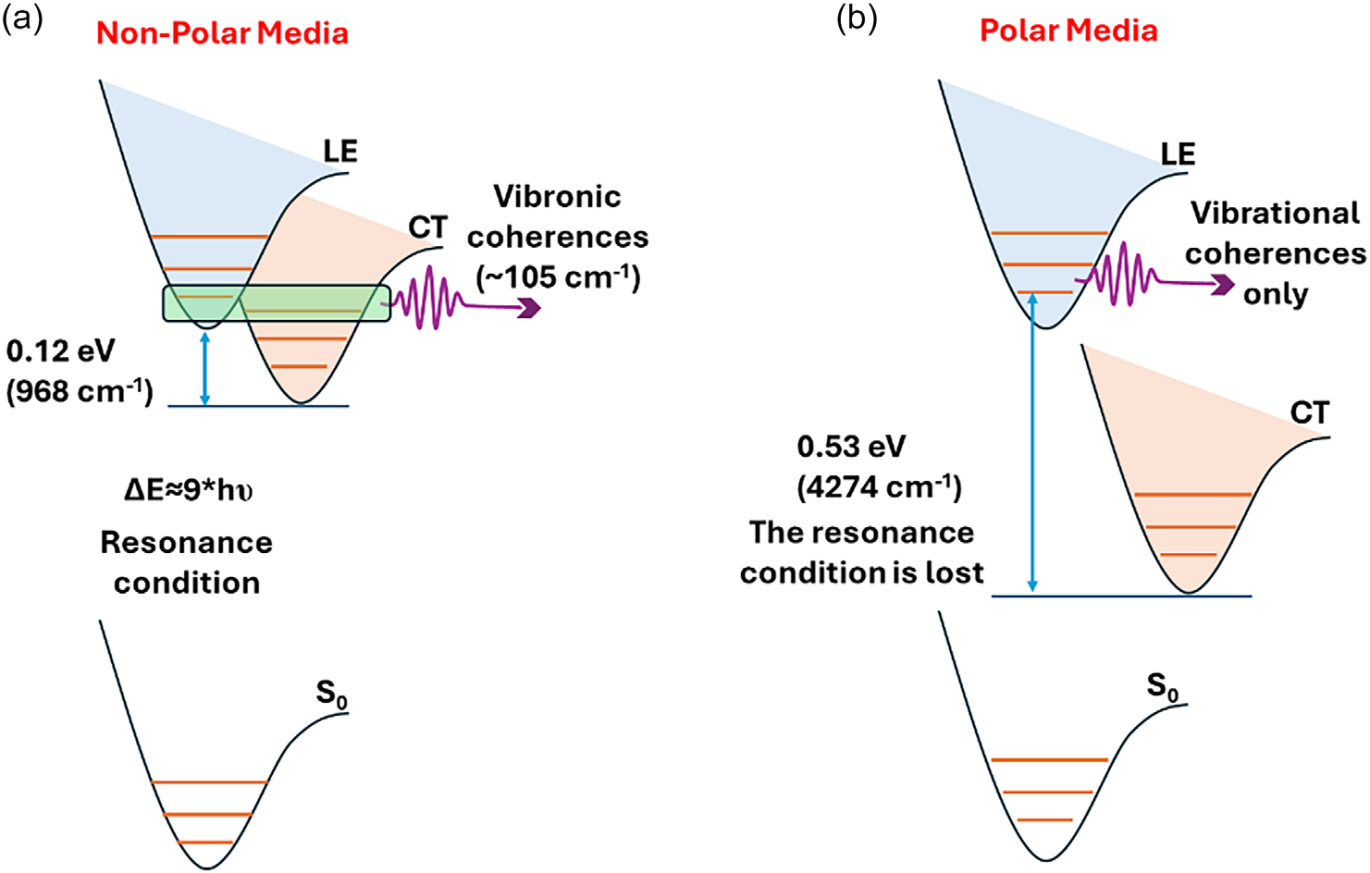
Resonance condition expected variations in polar and nonpolar media in D‐A systems with LE and CT coupled states. In n‐hexane, a near‐resonance condition is met, while in DCM the electronic gap is too large for resonance.

To understand the pronounced solvent‐dependent variation in the coherence behavior, we estimated the LE–CT energy separation in n‐hexane and DCM from the absorption/emission onsets in Figure S24 (Figure 6). Because the LE and CT bands overlap in n‐hexane, the LE energy was estimated from the DCM emission onset. In n‐hexane, the LE–CT gap is approximately 968 cm^−1^, while the dominant oscillatory frequency is about 105 cm^−1^. Under these conditions, the vibronic resonance condition (Δ*E* ≈ nhν) can be reasonably satisfied, here close to the *n* ≈ 9 condition, providing a clear basis for the strong vibronic coherences observed in the nonpolar medium [67]. In contrast, in DCM, the CT state is stabilized, and the LE–CT gap increases to approximately 4274 cm^−1^, which is expected to move the system away from resonance and suppress vibronic mixing. Consistent with this picture, the oscillatory features become significantly weaker or disappear in the polar solvent.

Importantly, even when individual LE and CT bands are not straightforward to assign with complete certainty, the solvent dependence of the oscillatory response remains highly informative. Our results indicate that vibronic coherences between LE and CT states are favored in nonpolar media, where the smaller LE–CT energy gap supports the resonance condition, and where the correspondingly smaller CT reorganization energy is expected to weaken system‐bath coupling and slow dephasing (see the caveat above regarding the relationship between polarity and reorganization energy), together supporting longer‐lived coherent motion. As solvent polarity increases, stabilization of the CT state widens the LE–CT gap, which is expected to increase the reorganization energy and strengthen dephasing, thereby quenching the resonance condition and diminishing the vibronic contribution. By contrast, vibrational coherences are expected to be much less sensitive to changes in the electronic energy gap and therefore to persist more robustly across solvents.

This solvent dependence therefore provides a practical and experimentally accessible way to distinguish vibrational from vibronic coherences using pump–probe spectroscopy alone. In nonpolar media, the LE–CT resonance condition is more readily met, and vibronic coherences are clearly observed, whereas in polar media, the resonance is progressively lost and the vibronic component is strongly damped. Vibrational coherences, on the other hand, remain comparatively insensitive to polarity and show far less change in lifetime or visibility. In this sense, solvent‐controlled coherence behavior offers a powerful diagnostic for identifying vibronic coupling without requiring temperature variation, which can complicate interpretation in TADF systems by shifting the electronic landscape itself. We note that this LE–CT mixing picture rests on adiabatic TDDFT calculations (transition energies, NTOs, and transition‐dipole orientations; see Supporting Information “Computational Studies”) together with the phase and solvent‐dependence evidence discussed above, rather than on an explicit diabatic‐state framework. This combination establishes a consistent, converging qualitative picture of LE–CT mixing, but does not, by itself, yield quantitative electronic coupling or mode‐resolved vibronic coupling constants. A diabatization procedure, for example, a fragment‐based or Boys/Generalized Mulliken‐Hush‐type localization scheme separating LE and CT diabatic states [68], would allow such quantities to be extracted directly, placing this picture on a more rigorous quantitative footing; we view this as an important and tractable direction for future computational work on this system, complementary to the experimental diagnostic developed here.

#### 2.2.4 Phase Relationship of the LE–CT Coherences

**FIGURE 7:**
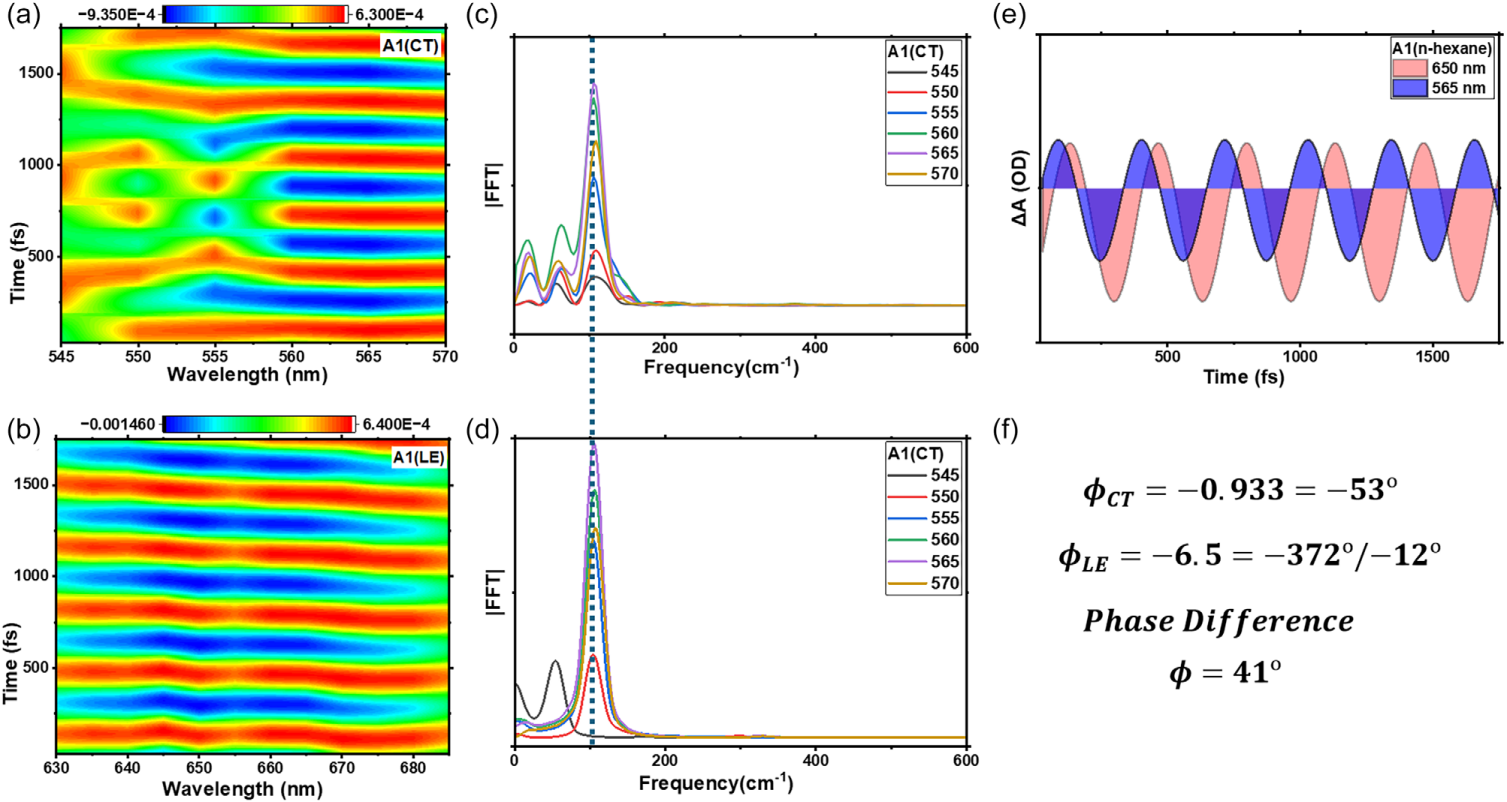
(a,b) Sine fit contours of the raw residual. (c,d) FFT of the sine fit contours shows a dominant frequency that is equal to the raw FFT of the residuals. (e) Sine wave of the two states on a particular probed wavelength and their phase. (f) Phase difference between the oscillations. (*All the color bars in contour plots show* Δ*A in OD* *.*).

To probe the phase relationship of the vibronic coherences, the residual oscillatory components associated with the LE and CT bands were fitted with a sine function (Figure S20). The corresponding sine‐wave contours for A1 are shown in Figure 7a,b, while the analogous results for A2 are provided in Figure S21. For the CT state, the fits were not optimal in the 540–555 nm region, although they were in reasonable agreement at longer wavelengths. By contrast, the LE‐band fits were good across the full spectral range. To verify that the fitted oscillatory component was physically present in the raw data, FFT analysis was performed on the sine‐fit residuals (Figure 7c,d). In both the LE and CT cases, the FFT revealed a dominant frequency near 105 cm^−1^, in agreement with the corresponding peak obtained directly from the FFT analysis of the raw data (Figure 5f,g). This agreement indicates that the fitted oscillation captures a real experimental component rather than a fitting artifact.

The extracted sine‐fit parameters are summarized in Table S4. For the CT band, the fitted phase is *φ*_CT_ = −0.9333 rad (∼−53°), whereas for the LE band it is *φ*_LE_ = −6.5 rad, which is equivalent to ∼−372° or ∼−12° after phase wrapping (Figure 7e). The phase difference between the LE and CT oscillations is therefore ∼41° for A1 and ∼10.8° for A2. These relatively small phase offsets indicate that the LE and CT coherences are nearly phase‐aligned, consistent with coherent coupling between the two components.

This experimentally determined phase relationship provides strong support for the interpretation that the intensity of TICT absorption is modulated by LE‐CT coupling. In this view, the observed phase behavior is consistent with partial intensity borrowing and constructive mixing of the LE and CT contributions, and it provides direct experimental evidence that the two oscillatory components interfere constructively. Accordingly, the relative phase appears to be a key factor in the enhanced TICT response observed in the UV/Vis spectra.

### 2.3 Section C

#### 2.3.1 Steady‐State and Time‐Gated Photoluminescence (SSPL/TGPL) Spectroscopy

**FIGURE 8:**
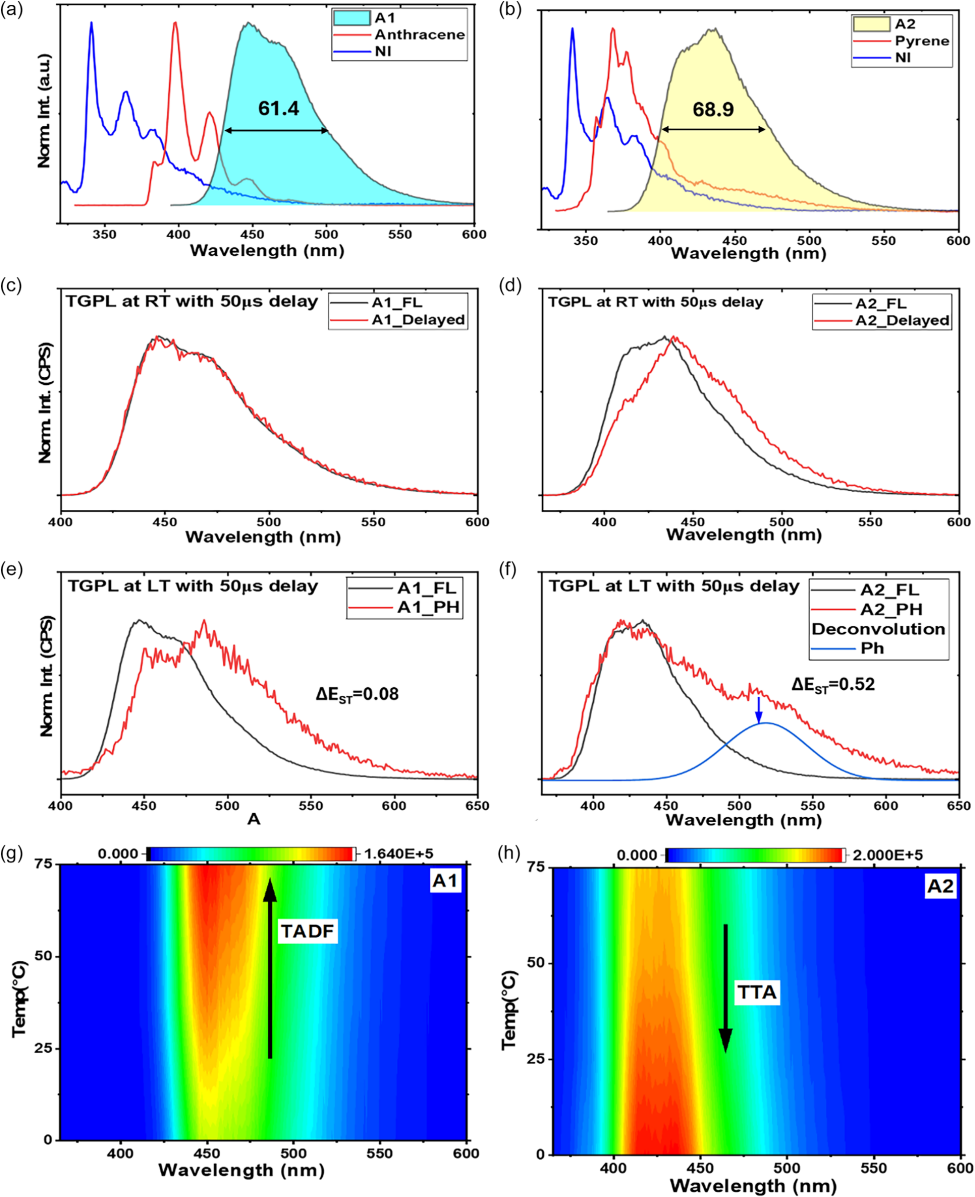
(a,b) SSPL spectra of A1 and A2 along with their respective donors (anthracene and pyrene, respectively) and the acceptor NI ring in n‐hexane. TGPL spectra with a 50 μs delay compared with SSPL and (c,d) RT and (e,f) LT; onsets of (b) are used to calculate the Δ*E*_ST_. (g,h) SSPL contours at varying temperatures exhibit opposite emission trends: A1 increases with temperature, while A2 decreases. (*The color bars in the contour plots show Intensity in a.u.*).

SSPL spectroscopy was performed in n‐hexane on the compounds, their respective donors, and the NI unit (Figure 8a,b) in n‐hexane. The emission of the compounds was isolated from the donor and acceptor units, indicating the CT nature of the emissive state. To confirm this, Solvatochromic studies were further performed (Figure S22). The emission redshifted drastically from 447 nm in n‐hexane to 600 nm in dimethylformamide (DMF) for A1 and from 436 to 581 nm for A2. In high‐polarity solvents like dichloromethane (DCM) and DMF, both compounds exhibited an additional band in the high‐energy region, attributed to local emission from donor and/or acceptor units. This confirmed the CT nature of the emissions. The compounds emitted in the blue region; thus, we determined their CIE coordinates in n‐hexane (Figure S23a,b). For A1, CIE_*x*_ = 0.145 and CIE_*y*_ = 0.122, which is the deep blue region, while A2 exhibited a pure blue color with CIE_*x*_ = 0.154 and CIE_*y*_ = 0.058.

To gain insight into the orbitals involved during emission, computational calculations were performed on the excited state. The CT excited singlet state was optimized, and confidence in the relaxed geometry was established by calculating vibrational frequencies, which yielded all positive values, confirming the true global minimum. The optimized geometry for *S*_1_ is provided in Figure S24. Notably, in the excited state, both A1 and A2 undergo severe planarization to the adiabatic state (donor and NI ring dihedral). A1 shows 58° while A2 shows 43° dihedral angles (Table S5). Then, we obtained the orbitals involved in emission (Figure S23c,d), and the oscillator strengths increased dramatically. A1 has *f* = 0.3204 and A2 possesses *f* = 0.7708 (Table S6). This is rationalized by increased planarization, as evidenced by the enhanced overlap between the electron and hole in the transition orbitals.

The TGPL experiments were conducted to investigate the compounds’ microsecond‐scale emissions. All the experiments were done in n‐hexane. First, we carried out time‐gated experiments at room temperature with a 50‐μs delay (Figure 8c,d). The spectra were compared to steady‐state fluorescence; the A1 TGPL spectrum matched the fluorescence, indicating its origin from the singlet state, while A2, along with singlet bands, exhibited an additional shoulder, likely due to a small room‐temperature phosphorescence (RTP) contribution. Thus, this delayed emission was attributed to DF, from TTA, TADF, or both. This delayed emission is attributed to the presence of a carbonyl on the NI ring, which allows the triplet state to be retrieved via DF (the NI ring also exhibits TTA characteristics; Figure S27). The NI acceptor exhibits DF emission (from LE transitions on the NI ring). DFT calculations using QChem software at ωB97XD/6‐311G(d,p) level of theory showed significant SOC between the singlet and triplet manifolds of NI, A1, and A2 (Table S7). Thus, we attribute the DF to carbonyl groups on the NI ring, which induce reasonable SOC and enable ISC and RISC to facilitate TTA/TADF.

Then, gated experiments were conducted at 77K with a 50‐μs time window to probe the triplet states of the molecules (Figure 8e,f). The compounds exhibited two bands: a lower‐energy band that redshifted relative to the fluorescence emission, and a high‐energy band that matched the steady‐state emission band. The lower‐energy band was attributed to phosphorescence emission from the triplet (*T*_1_) state, while the band corresponding to fluorescence was attributed to the *S*_1_ state due to DF. At 77K, the TADF mechanism cannot operate because of its thermal requirements; hence, the DF is likely attributable to TTA upconversion. Thus, both molecules show TTA upconversion. Further, the onsets were used to calculate the *T*_1_ and *S*_1_ energies (the redshifted peak of A2 was deconvoluted to obtain the *T*_1_ energy; Table 1). A1 shows a modest Δ*E*_ST_ of 0.08 eV, suggesting a possible TADF mechanism, while A2 has a larger ST gap of 0.52 eV, making TADF unlikely. The *T*_1_ energy of A1 redshifts substantially with increasing solvent polarity (2.87 eV in n‐hexane to 1.88 eV in DMSO), a Solvatochromic signature characteristic of CT states rather than an LE state. For A2, the particle‐hole character corroborates the triplet emission (Figure S26b), which shows charge redistribution from the NI‐centered orbital towards pyrene. We therefore assign the *T*_1_ states of both A1 and A2 to be predominantly ^3^CT in character.

Notably, in the TTA mechanism, two triplets form one higher singlet and another ground singlet. The energy ordering is important as twice the T_1_ energy (2E_T1_) should be greater than the *S*_1_ energy. In both cases, the 2E_T1_ are 5.9 and 6.38 eV for A1 and A2, respectively, which are higher than the S_1_ energies of both compounds. Thus, TTA is expected in both cases.

To confirm the DF mechanism, temperature‐dependent studies were conducted in n‐hexane (Figure 8g,h). Emissions were collected at varying temperatures. A1 exhibited gradual intensity enhancement with increasing temperature, attributed to a very small singlet‐triplet gap arising from a highly twisted geometry. At 0 °C, only the *S*_1_ molecules can be retrieved; however, as the temperature increases, thermal energy enables RISC, thereby enhancing overall emission. This is consistent with TADF behavior. In contrast, the A2 emission increased as the temperature decreased, attributed to reduced non‐radiative decay in the frozen matrix. Given a relatively large ST gap, the TADF mechanism is precluded, and the DF is assigned to TTA upconversion.

We have established DF; thus, triplet harvesting is possible. So, nitrogen/oxygen purging experiments were performed (Figure S28). Under aerated conditions (no O_2_/N_2_ purging), the quantum yield (QY) was about 0.44 for both A1 and A2. On purging nitrogen, the QY increased to 0.75 and 0.69 for A1 and A2, respectively. This is due to the presence of oxygen under aerated conditions, which quenches the triplet states. Once it is removed, the TTA/TADF mechanisms operate, contributing to overall fluorescence and ultimately increasing the QY. This demonstrates that both molecules are excellent triplet harvesters.

#### 2.3.2 TADF–TTA Switching and Polarity Robust Triplet Harvesting

**FIGURE 9:**
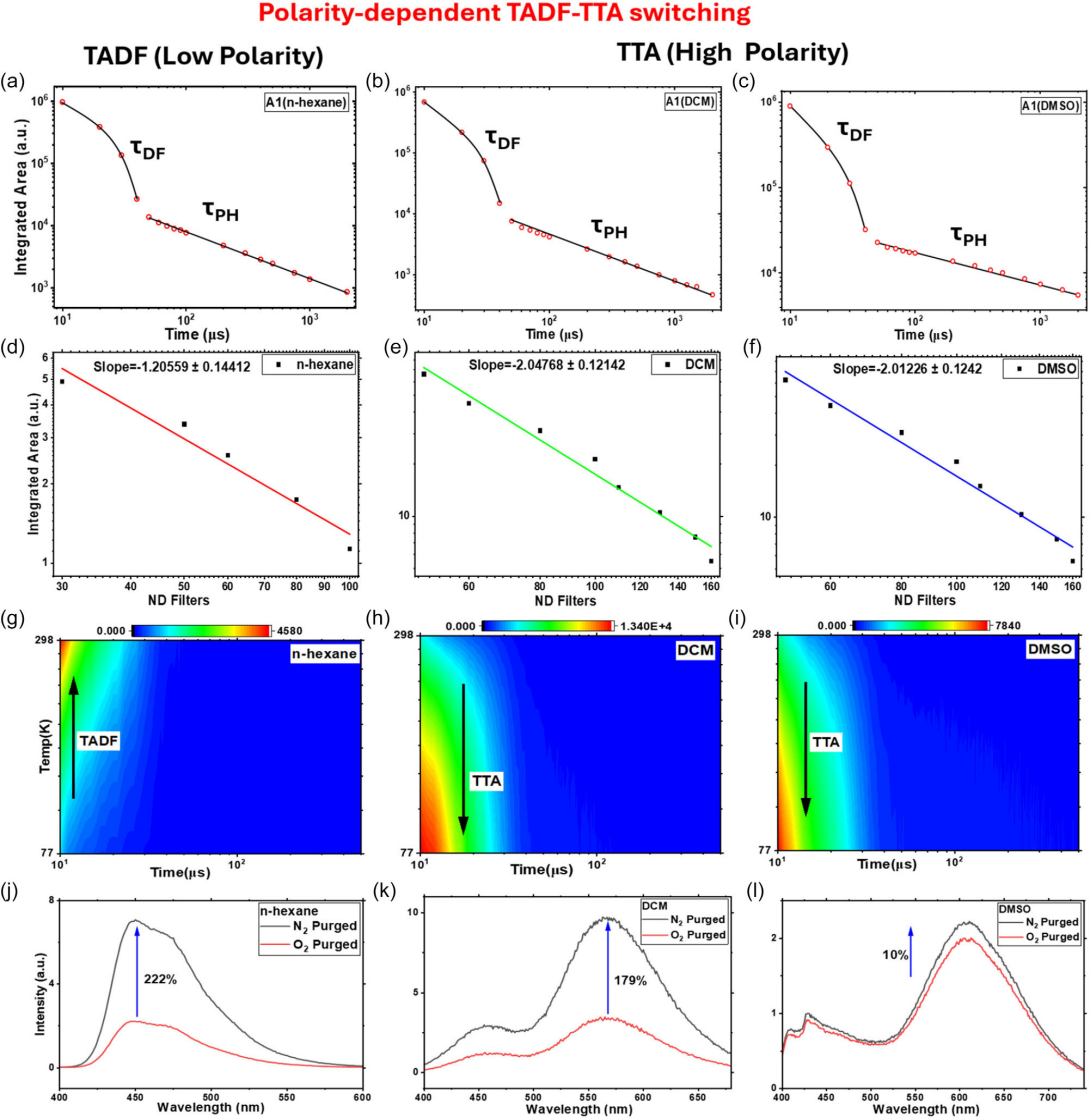
Photophysical evidence of polarity‐driven TADF‐to‐TTA switch in A1. (a–c) Time‐gated emission in various solvents as a function of the gated window shows two distinct components: the slow decay is attributed to DF (τ_DF_), while the longer component is assigned to phosphorescence (τ_PH_). (d–f) log–log spectra of emission as a function of excitation power show linear and quadratic slopes, confirming TADF and TTA, respectively. (g–i) TRPL contours at low and high temperatures further support TADF and TTA behaviors in different polarities. (j–l) Steady‐state emission enhancement on nitrogen purging shows the triplet harvesting efficiency and indicates a significant triplet population. (*The color bars in contour plots show Intensity in a.u.*).

Now, we move on to our last objective: switch between TADF and TTA as a function of polarity in A1. First, we performed time‐gated experiments in n‐hexane, DCM, and DMSO with a minimum delay of 10 μs (Figure 9a–c). The integrated area as a function of delay time shows two distinct components: the short‐lived component decays within 50 μs, while the long‐lived component persists up to 2 ms. The short‐lived component was attributed to DF, while phosphorescence was mainly responsible for the long‐lived component.

To understand the origins of this DF in each case, we conducted excitation power‐dependent experiments. Figure 9d–f presents log–log plots of integrated TADF emission area versus excitation power density, providing critical mechanistic insight into the DF pathways. Neutral density (ND) filters were applied, with lower values indicating fewer photons are cut off and higher values indicating more photons are cut off. For n‐hexane, we observe linear scaling with a slope of ~1 (red trace). This confirms the TADF mechanism: the emission intensity scales directly with the single‐photon triplet generation rate, followed by thermally activated RISC to the emissive singlet state. In contrast, DCM and DMSO exhibit a quadratic scaling with slopes ≈2 (green/blue traces), indicating TTA: a higher‐order process in which emission scales with the square of the triplet density, as two triplets collide to produce one emissive singlet. This is expected as the ST gap is considerably large in DCM and DMSO for the RISC process in these two polar environments. These distinct power dependences rigorously distinguish between TADF and TTA. To further confirm this TADF‐TTA switching, we performed time‐resolved PL (TRPL) spectroscopy at varying temperatures (Figure 9g–i). The results unambiguously show that the delayed time‐resolved components increase with temperature in n‐hexane but decrease for DCM and DMSO. This is consistent with TADF and TTA behavior, respectively. Thus, we see TADF‐TTA switching in A1 as the solvent polarity increases.

At this point, it should be noted that TTA is commonly observed in many organic molecules; the challenge is to obtain a significant triplet population that can be retrieved, and only then will the TADF–TTA switching mechanism be important. To obtain triplet contributions across various environments, we performed nitrogen/oxygen purging experiments (Figure 9j–l). Under oxygenated conditions, the triplet is expected to be quenched, leaving only singlet emission. As soon as nitrogen is purged, the triplet state is expected to contribute to the overall emission. The results show a 222% increase in n‐hexane emissions, a 179% increase in DCM, and only a 10% increase in DMSO. Inefficient triplet harvesting via TTA in DMSO is attributed to its high viscosity, which drastically reduces TTA efficiency. In the previous two cases, 222% and 179% increases are substantial and indicate that the triplet population is significant. This is of vital importance, as TADF is expected to quench markedly with increasing solvent polarity (as corroborated by Δ*E*_ST_ in DCM), thereby hindering the flexibility of device fabrication. In fact, triplet harvesting should be extremely inefficient in polar environments if the molecule exhibits a TADF‐only mechanism. This suggests an advantage of TADF–TTA switchable probes. This can provide greater flexibility in selecting polymer matrices for OLED device fabrication and in optimizing overall device performance. We note that this advantage is inferred from solution‐phase photophysical characterization; we have not yet fabricated or tested actual OLED devices based on A1 or A2, and the device‐level performance implications discussed here should therefore be regarded as a motivated hypothesis rather than an experimentally validated outcome.

## 3 Conclusion

In summary, this work establishes a solvent‐polarity‐based pump‐probe strategy for distinguishing vibronic from vibrational coherences in D‐A systems with LE–CT mixing, thereby addressing a long‐standing limitation in the interpretation of ultrafast coherent dynamics. In a nonpolar medium, the small LE–CT energy gap and weaker system‐bath coupling support resonant vibronic mixing and persistent oscillations across both LE and CT ESA bands, whereas in a polar medium, CT stabilization enlarges the LE–CT gap, accelerates dephasing, and suppresses the vibronic contribution while leaving vibrational coherence comparatively more robust. This provides a practical diagnostic that avoids the ambiguities of temperature‐dependent approaches in TADF systems, where conformational and energetic changes can themselves obscure coherence assignment.

More importantly, the present results show that this distinction carries broader photophysical significance. The identification of vibronic LE–CT coupling reveals that the optical response of twisted D‐A chromophores is governed not only by geometry but also by state mixing, phase relation, and intensity borrowing. This explains why CT/TICT absorption can remain visible even in highly twisted systems and why apparently similar 1,8‐naphthalimide derivatives can exhibit either bright or quasi‐dark low‐energy absorption. In this way, coherence analysis becomes a mechanistic probe of LE–CT hybridization and provides a qualitative basis for understanding the effective CT transition dipole and its role in enhancing or suppressing absorption strength.

The same D‐A framework also dictates how triplets are harvested. By demonstrating polarity‐controlled switching between TADF and TTA in a single deep‐blue emitter, this study shows that environment‐dependent LE–CT energetics can redirect the delayed‐emission pathway while preserving significant triplet utilization. This is important because conventional CT‐based TADF often becomes less effective in polar environments, whereas a TADF–TTA switchable architecture can retain functionality by accessing a complementary triplet‐harvesting channel. Taken together, these results connect coherence physics, CT absorption, and delayed‐emission switching into one unified picture and provide a broader guideline for designing efficient, polarity‐robust donor–acceptor materials for optoelectronic applications.

## Conflicts of Interest

The authors declare no conflicts of interest.

## Supporting information

Supporting information (SI) contains experimental details (materials, instrumentation, synthesis, and characterizations) and methodology. The results section of the SI presents data from DFT, UV/Vis, steady‐state/time‐gated PL, time‐resolved PL, and ultrafast TAS.

## Data Availability

The data that support the findings of this study are available in the supplementary material of this article.
