# Supplementary material for: Solvent Polarity as a Diagnostic for Vibronic/Vibrational Coherences Distinction in an Efficient TADF‐TTA Switchable Emitter
Source: Small Sci. 2026 Aug 21;6(8):e70387. doi: 10.1002/smsc.70387 (PMC13517017; doi:10.1002/smsc.70387)
Supplement: Supplementary file 1 [file SMSC-6-e70387-s001.pdf]

Electronic Supplementary Information

Solvent Polarity as a Diagnostic for Vibronic/Vibrational Coherences Distinction in an Efficient TADF-TTA Switchable Emitter

Ajeet K. Singh[a], M Sridevi[b], Dhrubajyoti Paul[a], Annu Agarwal[a], Nancy Punia[a], Rajiv Singh [b*], Inamur Rahaman Laskar [a*]

[a] Mr. Ajeet K. Singh, Mr. Dhrubajyoti Paul, Ms. Annu Agarwal, Ms. Nancy Punia, and Prof. Inamur Rahaman Laskar
Department of Chemistry, Birla Institute of Technology and Science, Pilani, Pilani Campus, Vidya Vihar, Pilani, Rajasthan, 333031, India.
E-mail: ir_laskar@pilani.bits-pilani.ac.in

[b] Ms. M Sridevi and Prof. Rajiv K Singh
Photovoltaic Metrological Section, Advanced Materials & Device Metrology Division, CSIR-National Physical Laboratory, Dr. K.S. Krishnan Marg, New Delhi-110012, India.
E-mail: rajivsingh.nplindia@csir.res.in

Experimental Section:

Materials:

We have synthesized proposed compounds A1 & A2. 4-bromo-1,8-naphthalic anhydride was procured from BLD Pharma, and 4-fluoroaniline was obtained from Spectrochem. The UV-grade solvents methanol, hexane, toluene, dichloromethane, and DMSO were supplied by Spectrochem. 9-Anthraceneboronic acid, pyrene-1-boronic acid, and palladium (0) were purchased from TCI. Potassium carbonate was obtained from Merck. All the compounds were used without further purification.

Instrumentation

Structural Characterizations, Steady-state, and Time-gated Photophysics

A 400 MHz Bruker NMR spectrometer was used to record ^1H and ^{13}C NMR spectra. UV-vis absorption spectra were measured on Shimadzu UV-1800/2550 spectrophotometers (solution) and UV-2450 (solid-state, absorption mode). Steady-state and time-gated PL used Horiba FluoroMax+ and FluoroLog spectrofluorometers. Time-resolved PL employed a gated iCCD camera (Stanford Computer Optics), excited by 355 nm (third harmonic of Nd: YAG laser). Temperature-dependent measurements (273-343 K) were performed using a Janis Research LN_2 cryostat. PL quantum yields were determined with a Hamamatsu Quantaurus-QY system.

Ultrafast Spectroscopy

The ultrafast optical pulses were generated using a Coherent Micra Ti: sapphire laser amplifier operating at 800 nm, with a pulse duration of 35 fs, a pulse energy of 4 mJ, and

a repetition rate of 1 kHz. The output beam was split into a pump and a probe beam using a 70:30 beam splitter. The higher-energy beam was directed into a Light Conversion TOPAS-C optical parametric amplifier to generate tunable pump pulses spanning 190-2600 nm. The weaker beam was focused on a sapphire crystal to produce a white-light continuum, which served as the probe. The pump and probe beams were spatially overlapped at the sample position. A mechanical delay stage introduced a temporal delay between the pump and probe pulses, enabling transient measurements over a delay window of up to 6 ns.

Methodology:

Computational Studies

Gaussian 16 (visualized in GaussView)[1] was used for all computations. Ground-state (S_0) and excited-state (S_1) geometry optimizations, TDDFT absorption and emission calculations, and HOMO-LUMO energies reported throughout this work (**Figure 1g-i, Figure 24S, Table 1S, Table 2S, Table 3S, Table 6S**) were obtained at the cam-B3LYP/6-311+G(d,p) level of theory, using Pople's 6-311+G(d,p) basis set, with added polarization/diffuse functions for electronegative atoms.[2] Quadratic SCF convergence was employed throughout. All calculations were performed in the gas phase, without an implicit solvent model (e.g., PCM); solvent-dependent trends discussed in the main text are based on experimental spectroscopic data (absorption/emission onsets) rather than solvent-corrected calculations, and the reported geometries and TDDFT transition energies should be interpreted as gas-phase values.

Photophysical studies

All the UV/Vis, steady-state, and time-gated spectroscopies were performed using 10^{-5} M solutions. Excitation power time-gated spectroscopy was performed using neutral density (ND) filters ranging from 10 to 300. A lower ND value indicates fewer photons are cut, while a higher value indicates weaker excitation power.

Ultrafast Spectroscopy

For ultrafast transient absorption (UTAS) measurements, samples A1 and A2 were separately dispersed in dichloromethane (DCM) and n-hexane, respectively, and sonicated for 30 min to ensure homogeneous dispersion. The solutions were then left undisturbed for 15 min to allow larger particulates to settle. Subsequently, the clear supernatant was carefully collected and used for the transient absorption measurements. UTAS measurements were performed on both samples in both solvent media. The samples were excited with a 350 nm pump pulse at an average power of 0.5 mW, and the transient response was probed in the visible spectral region. For each sample, two

sets of transient absorption spectra were recorded. The first dataset was acquired over a short delay window of up to 100 ps, with a higher density of delay points to resolve early-time ultrafast processes and coherent oscillatory dynamics with improved temporal resolution. The second dataset was recorded over an extended delay range of up to 6 ns, with a lower density of delay points, to monitor the longer-timescale excited-state relaxation and decay behavior.

Schemes 1 and 2: Synthetic route of A1-A2

Br-NA-nBz-F (Yield= ~90%):

1 eq. of 6-Bromobenzo[de]isochromene-1,3-dione and 1.5 eq. 4-fluoroaniline was taken in a two-neck round-bottom (RB) flask. 4.2 mL of methanol solvent was added to the above mixture. The reaction was refluxed at 70℃ for 12 hours under nitrogen. The reaction was brought to room temperature (RT), filtered, and washed with methanol. The ethanol was removed by suction filtration, and the solid was dried under vacuum for 4 h to obtain a yellow-brown solid.

A1 (Yield= ~64%):

1 eq. of the Br-NA-Nbz-F was taken in a two-neck RB flask, and 1.5 eq. of 9-Anthraceneboronic acid was added. The setup was under a nitrogen atmosphere, and toluene was used as the solvent. 10% of $Pd(PPh_3)_4$ was added with 1.5 eq. of K_2CO_3; the reaction was refluxed for 18 hours. The reaction was brought to RT and worked up three times with ice-cold water, separating the ethyl acetate and water layers each time. A compound mixture was obtained; the compound was separated by column chromatography using a 15% ethyl acetate/hexane mixture. The pure product was obtained and dried under vacuum using a rotary evaporator. HRMS, ^{1}H-NMR, and ^{13}C-NMR were used to characterize the structures of the compounds.

A2 (Yield= ~55%):

1 eq. of the Br-NA-Nbz-F was taken in a two-neck RB flask, and 1.5 eq. of pyren-1-ylboronic acid was added. The setup was under a nitrogen atmosphere, and toluene was used as the solvent. 10% of $Pd(PPh_3)_4$ was added with 1.5 eq. of K_2CO_3; the reaction was refluxed for 18 hours. The reaction was brought to RT and worked up three times with ice-cold water, separating the ethyl acetate and water layers each time. A compound mixture was obtained. The compound was separated by column chromatography using a 15% ethyl acetate/hexane mixture. The pure product was obtained and dried under vacuum using a rotary evaporator. HRMS, ^{1}H-NMR, and ^{13}C-NMR were used to characterize the structures of the compounds.

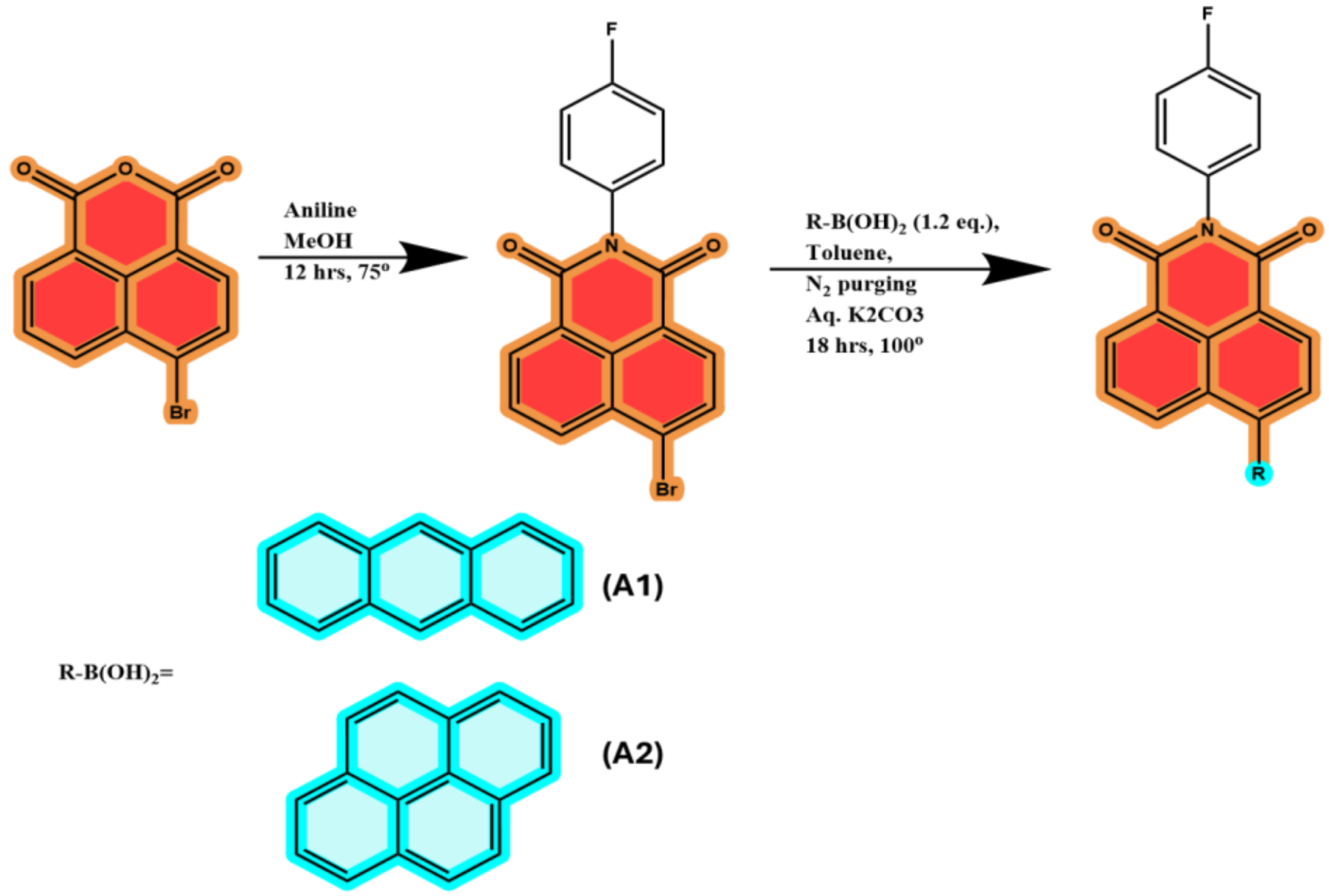

Scheme 1: Synthetic route of A1 and A2

Structural Characterizations

A1

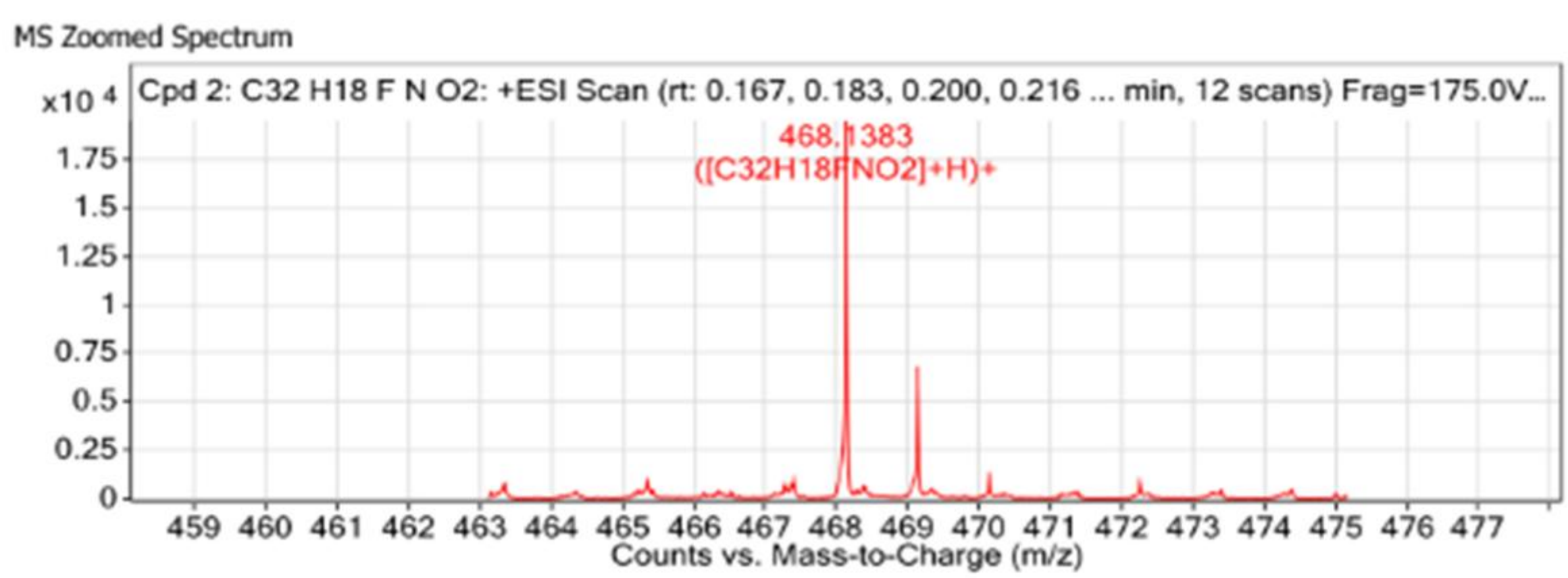

Figure 1S: HRMS of A1

1H NMR (400 MHz, Chloroform-d) δ 8.88 (d, J = 7.4 Hz, 1H), 8.70 (d, J = 2.8 Hz, 1H), 8.17 (d, J = 8.5 Hz, 2H), 7.90 (d, J = 7.4 Hz, 1H), 7.53 (dq, J = 6.8, 3.5, 2.8 Hz, 4H), 7.40 (dd, J = 8.6, 4.9 Hz, 2H), 7.36 – 7.26 (m, 7H).

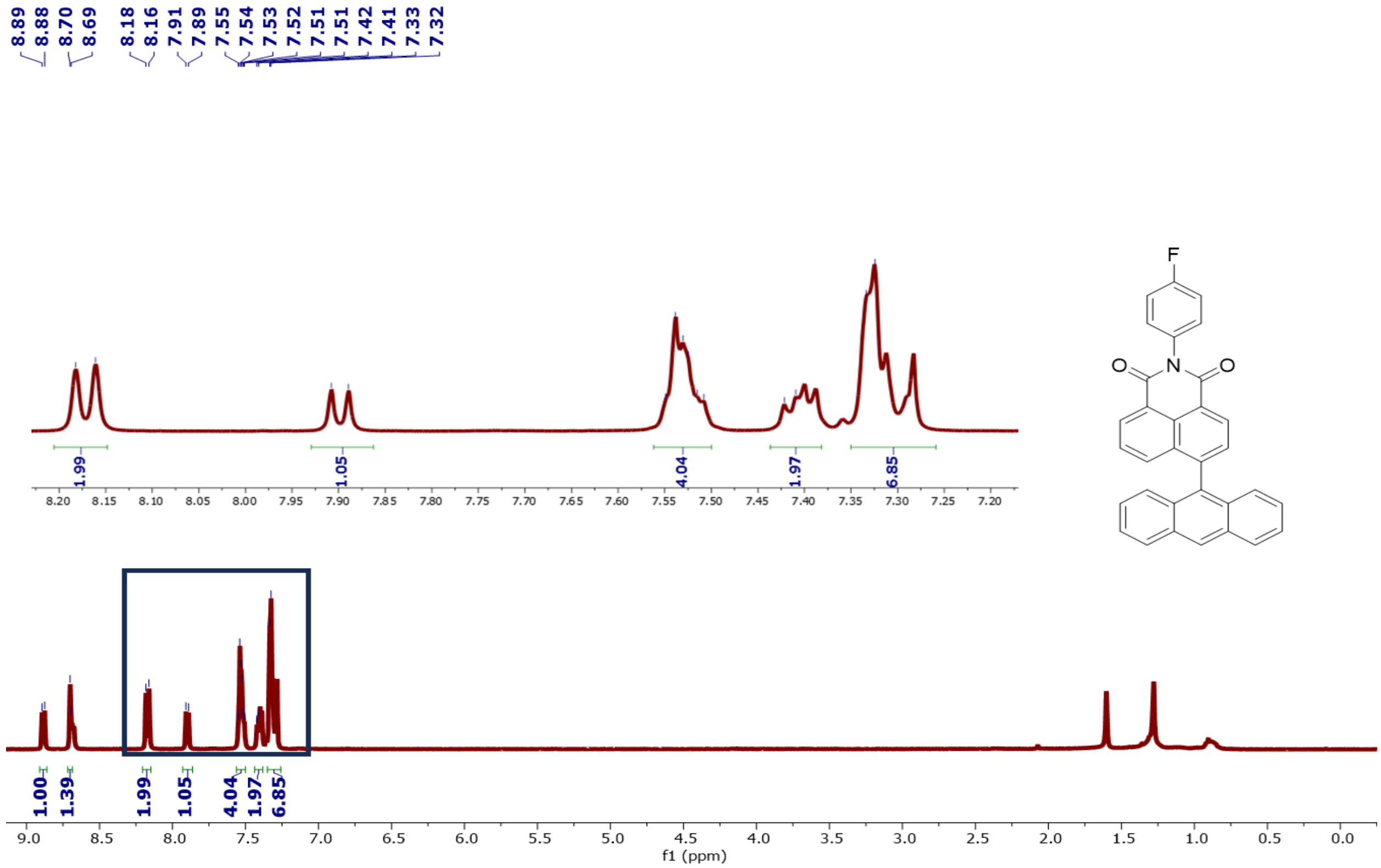

Figure 2S: 1H NMR of A1

^{13}C NMR (101 MHz, Chloroform-d) δ 164.51, 164.41, 163.79, 161.33, 144.75, 133.44, 132.16, 132.09, 132.03, 131.35, 131.27, 130.59, 130.54, 130.45, 130.40, 128.92, 128.74, 128.19, 127.37, 126.38, 126.02, 125.49, 122.97, 122.52, 116.65, 116.42.

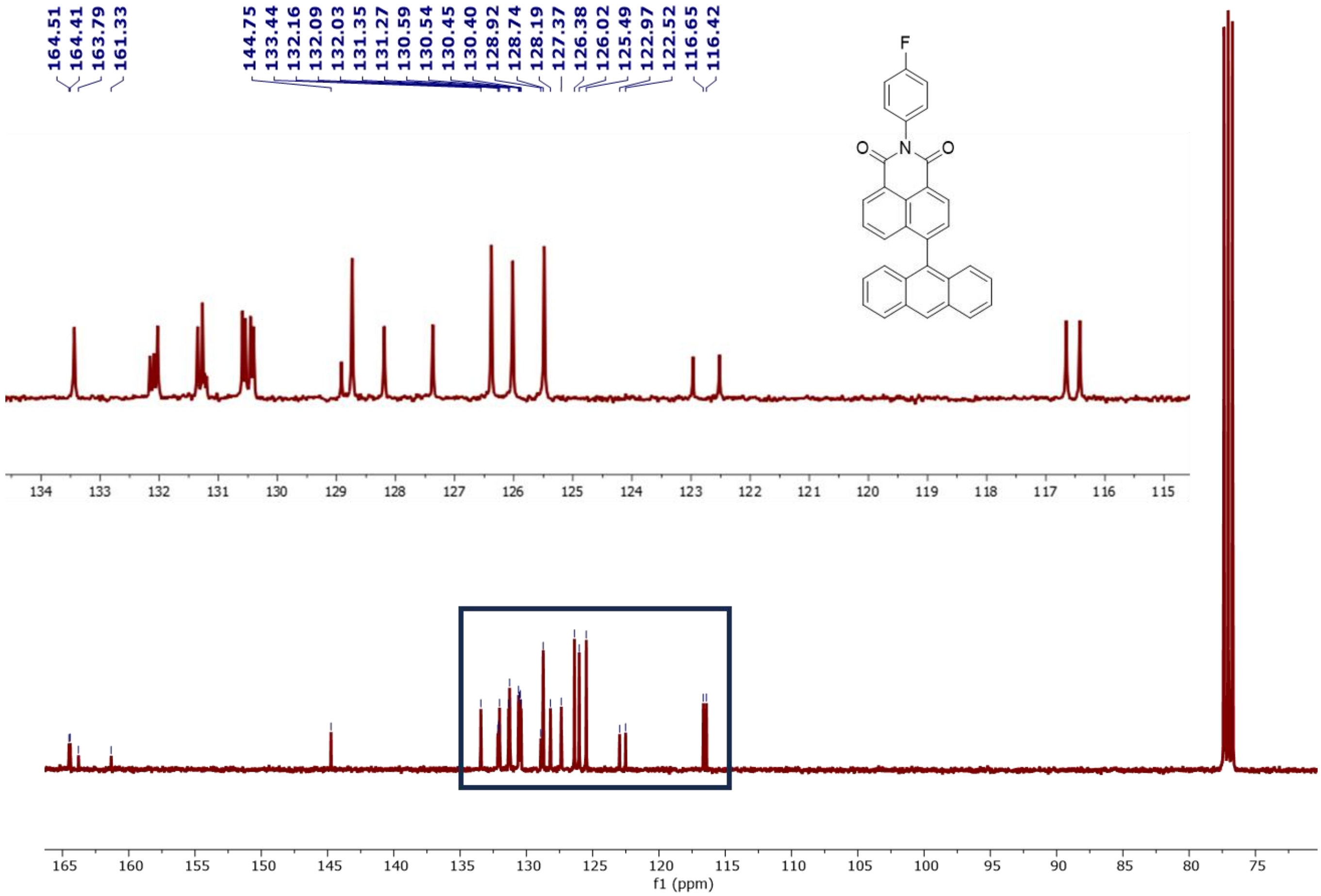

Figure 3S: 13C NMR of A1

A2

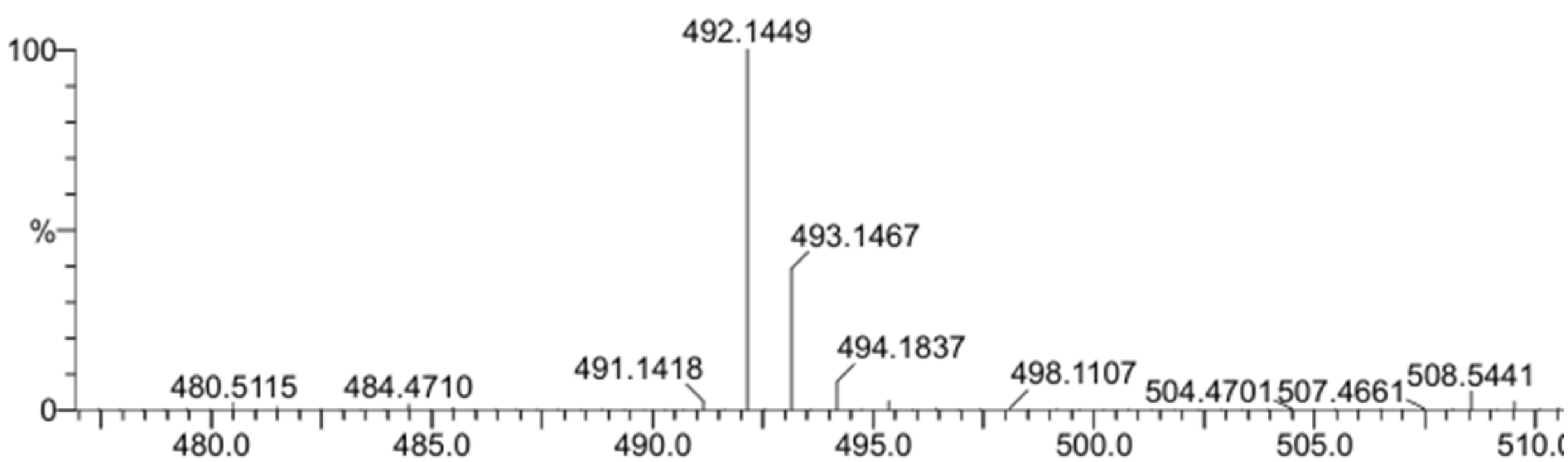

Figure 4S: HRMS of A2

^{1}H NMR (400 MHz, Chloroform-d) δ 8.71 (dd, J = 7.3, 1.2 Hz, 1H), 8.66 (dd, J = 8.4, 1.2 Hz, 1H), 8.47 (d, J = 7.8 Hz, 1H), 8.10 (d, J = 7.8 Hz, 1H), 7.91 (dd, J = 8.5, 7.3 Hz, 1H), 7.36 – 7.21 (m, 4H).

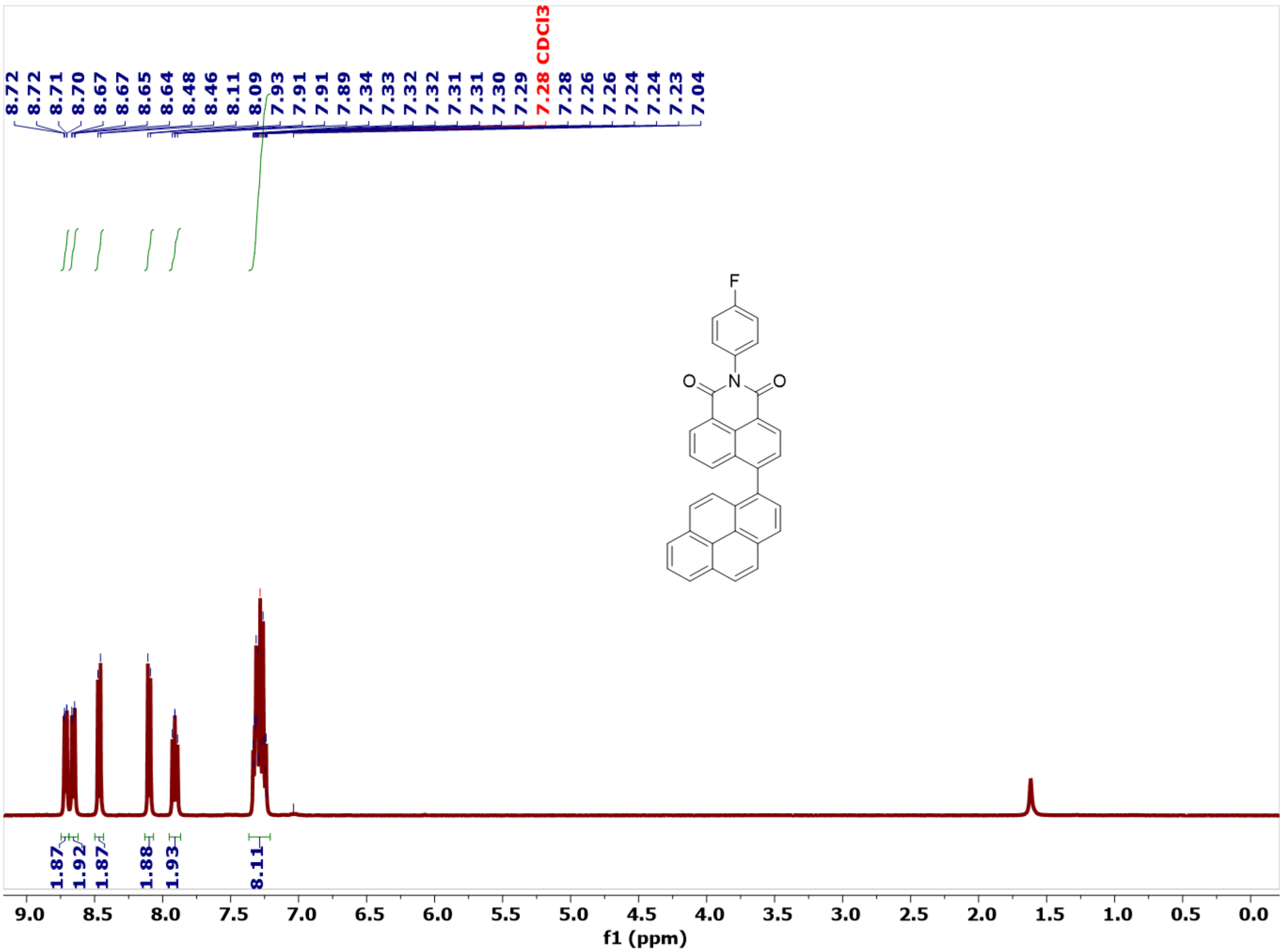

Figure 5S: 1HNMR of A2

^{13}C NMR (101 MHz, Chloroform-d) δ 164.54, 164.39, 133.51, 133.32, 133.15, 132.11, 131.71, 131.44, 131.28, 131.11, 130.81, 129.59, 128.31, 127.87, 127.66, 127.60, 127.35, 127.15, 126.45, 126.08, 125.83, 125.54, 124.79, 124.61, 123.10, 122.44.

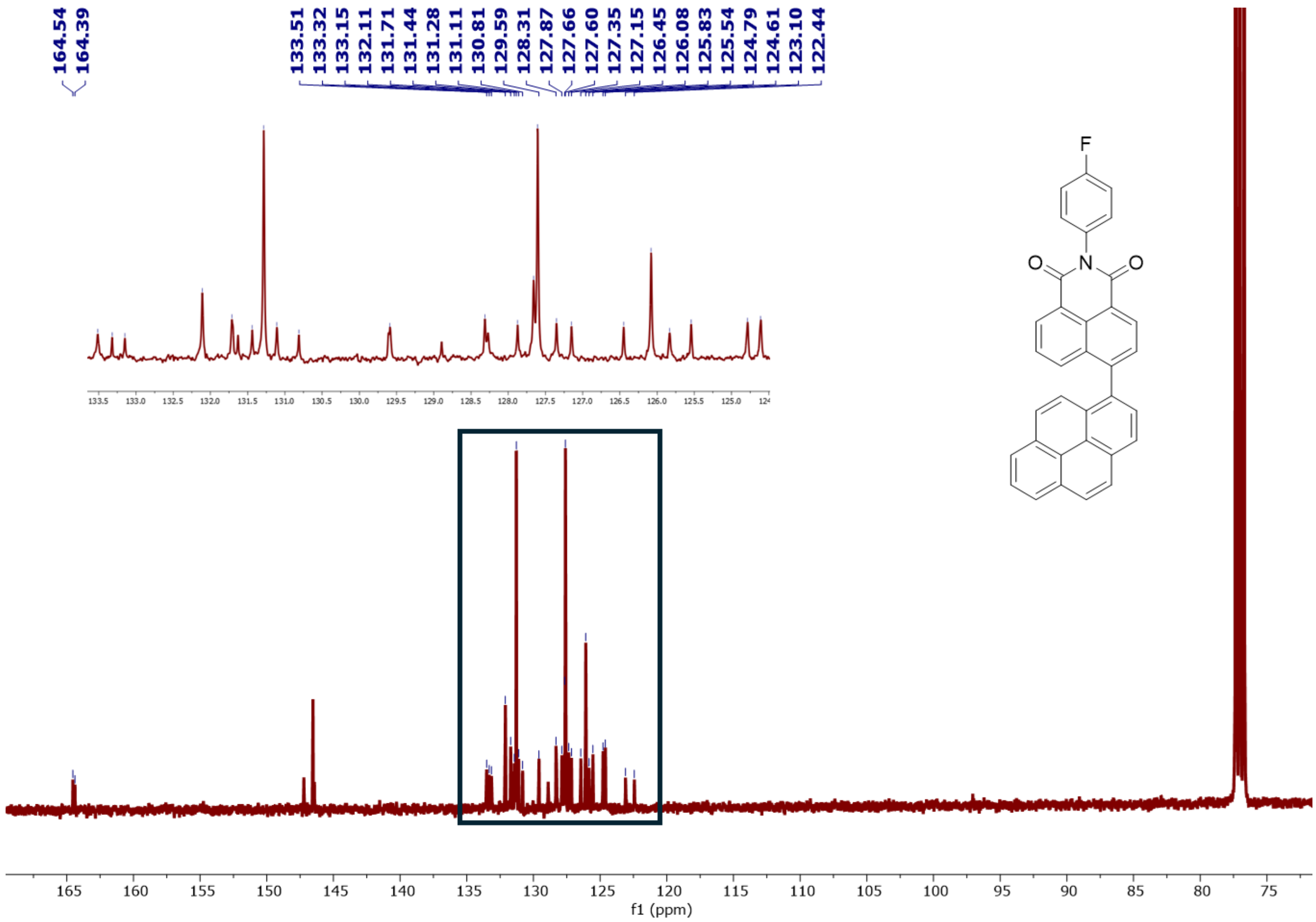

Figure 6S: 13CNMR of A2

Theoretical Framework

Consider a three-state model comprising two excited states (**Figure 1d**): S_1, predominantly CT character, and a state S_2, predominantly LE, in addition to the ground state (S_0). The TDM of these individual states under the Frank-Condon regime can be written as

$$\mu_{S1} = \langle \psi_{S1} | \overrightarrow{\mu_{S1}} | \psi_{S0} \rangle$$ **Equation A**

$$\mu_{S2} = \langle \psi_{S2} | \overrightarrow{\mu_{S2}} | \psi_{S0} \rangle$$ **Equation B**

When LE and CT states couple, the effective transition dipole moment (TDM) of the resulting S_1 state is a linear combination of the individual contributions, with mixing coefficient α scaling with the coupling strength.[3] The effective TDM, μ_{S1}^{eff} , is expressed as:

$$\mu_{S1}^{eff} = \mu_{S1} + \alpha\mu_{S2}$$ **Equation C**

To consider the direction of TDMs

$$\mu_{S1}^{eff} = \mu_{S1} + \alpha * \cos\theta * \mu_{S2}$$ **Equation 2**

Equation 2 accounts for TDM orientation: θ is the angle between mixed S_1 and S_2 TDMs. LE TDM remains fixed for specific D/A units, while CT TDM usually points from donor to acceptor.

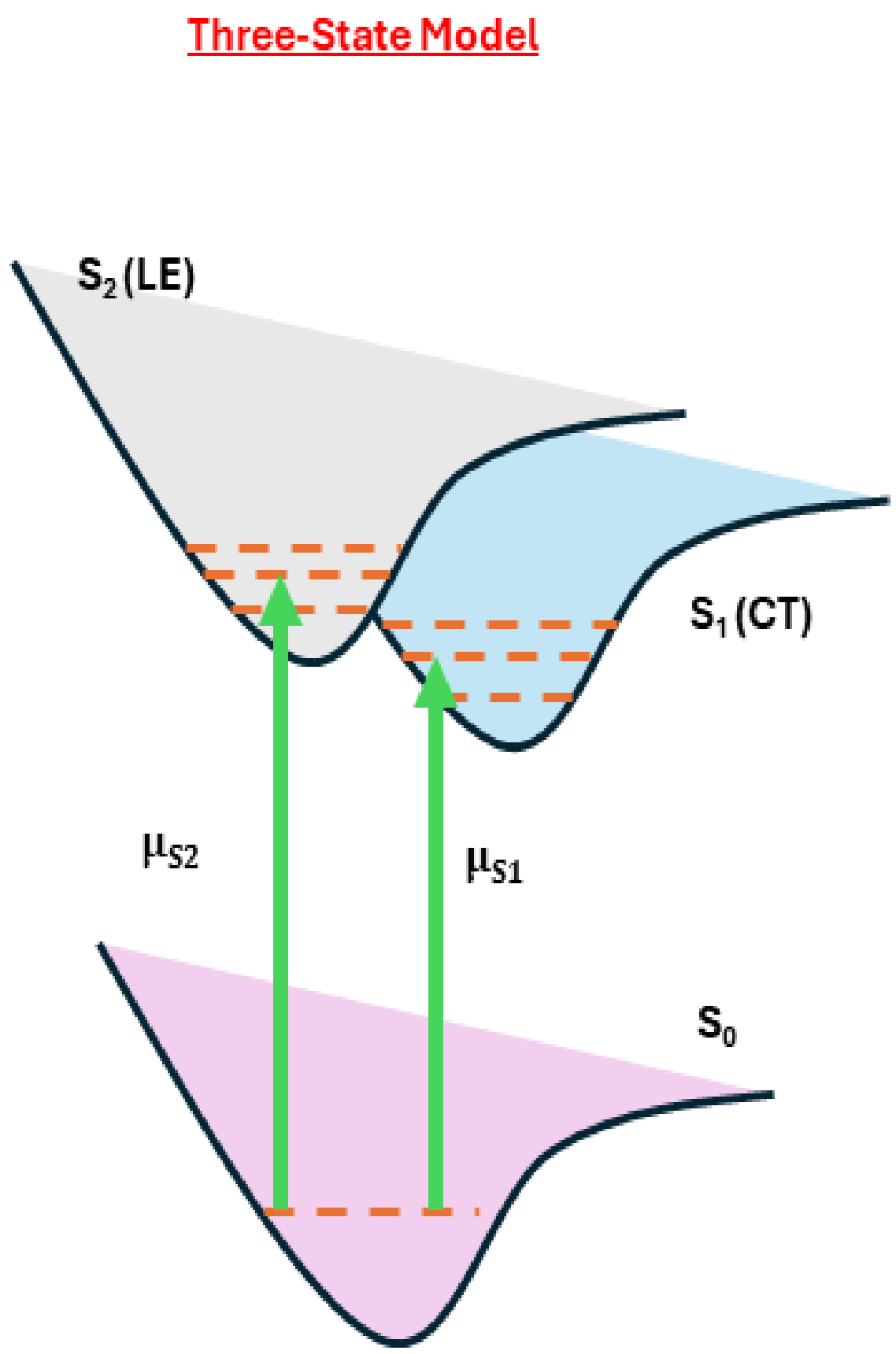

$$\mu_{S1}^{eff} = \mu_{S1} + \alpha * \cos\theta * \mu_{S2}$$

Figure 7S: The three-state model in mixed states, with S1 having CT character predominantly, while S_2 has LE characteristics.

Results and Discussions

UV/Vis Spectroscopy

Computational Details

Table 1S: HOMO-LUMO energies (in eV) of the molecules in the ground state.

Compound	HOMO	LUMO
**A1**	-7.63	-1.06
**A2**	-7.66	-1.06

Table 2S: Dihedral angle between donors (anthracene and pyrene) and the NI ring of the two compounds in the ground state.

Compound	Dihedral angle (donor/NI ring)
**A1**	89.71°

**A2**	72.10°
**A3**	84.28°

Table 3S: TDDFT details during absorption of the molecules depicting the energies, CT transitions, and their respective oscillator strength.

Compound	Energy (eV)	Nature of transition	Oscillator strength (f)
**A1**	3.79	CT	0.0002
**A2**	3.84	CT	0.6585
**A3**	3.81	CT	0.0005

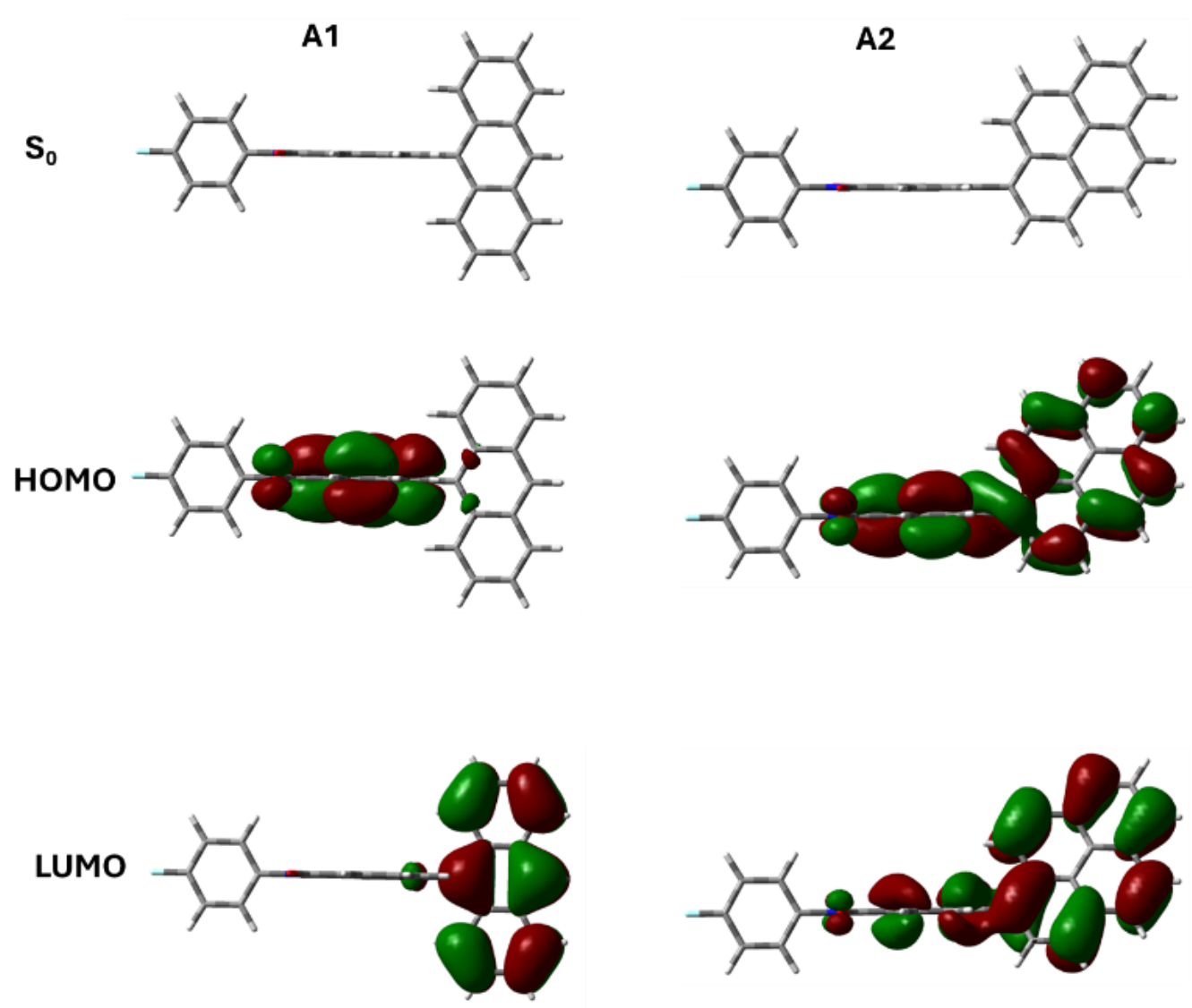

Figure 8S: Ground state geometry and HOMO-LUMO orbital overlap of A1 and A2.

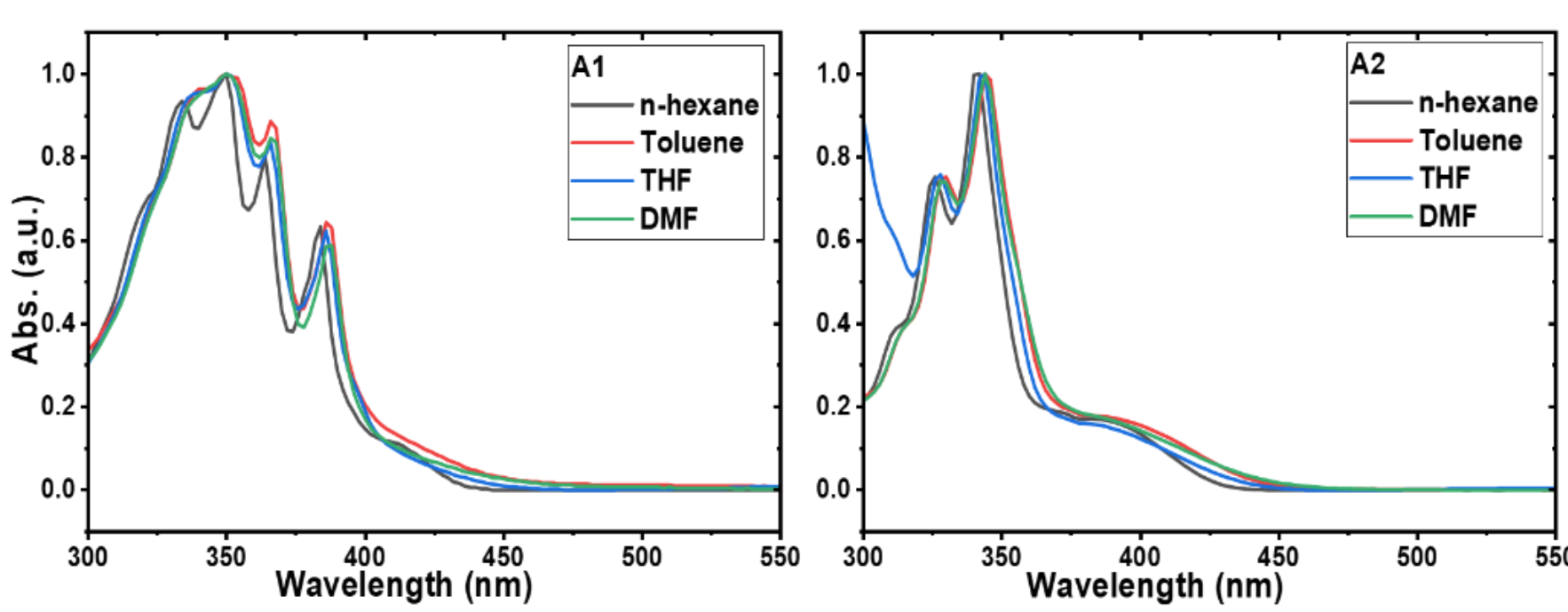

Figure 9S: Solvatochromic absorption spectra of the molecules in various solvents showing minimal changes.

Ultrafast Transient Absorption Spectroscopy:

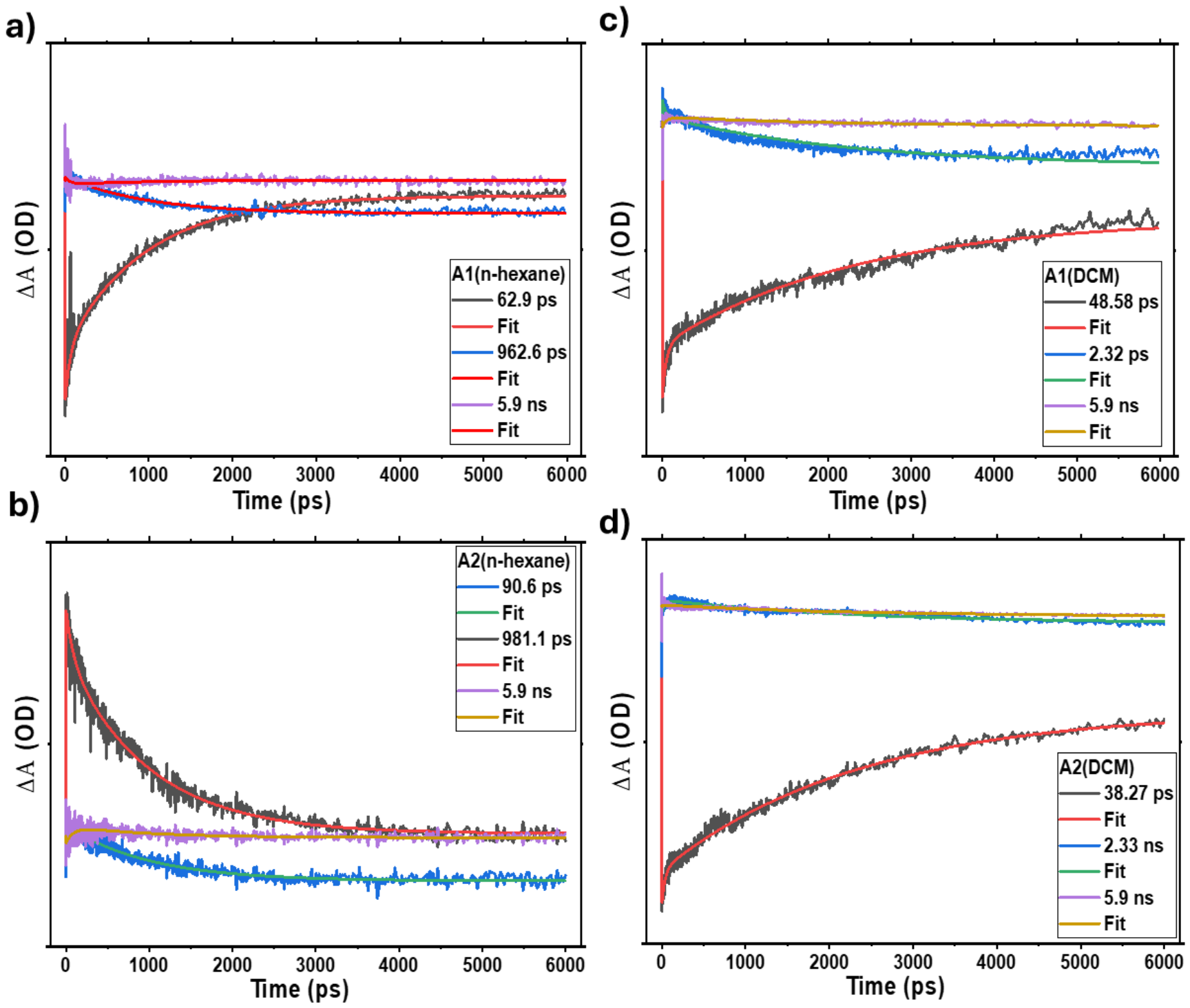

Figure 10S: The global kinetics of EADS components in **(a-b)** n-hexane and **(c-d)** DCM.

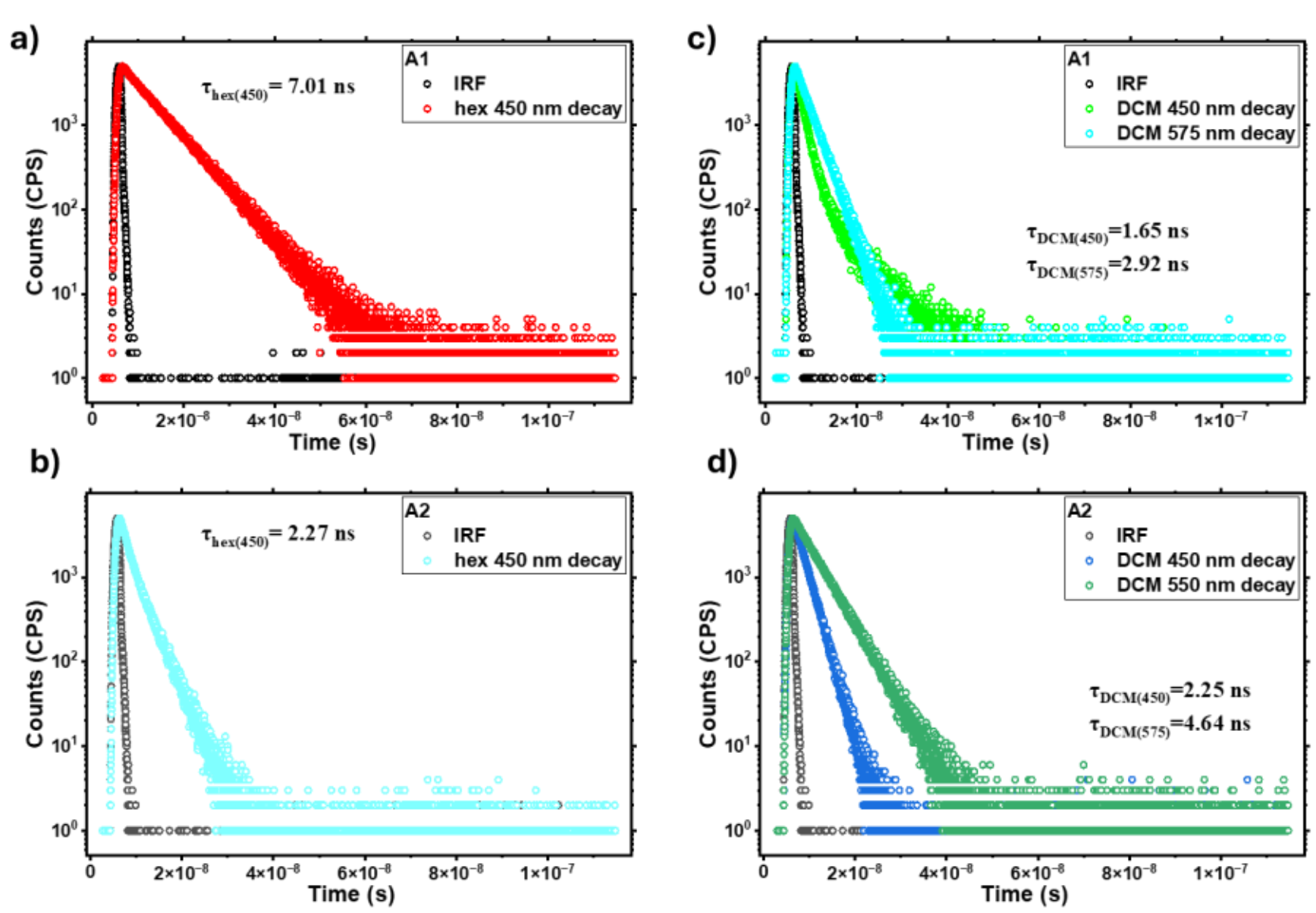

Figure 11S: Steady-state lifetime of the excited states using a 370 nm excitation source in n-hexane and DCM.

Vibrational Vs. Vibronic Coherences

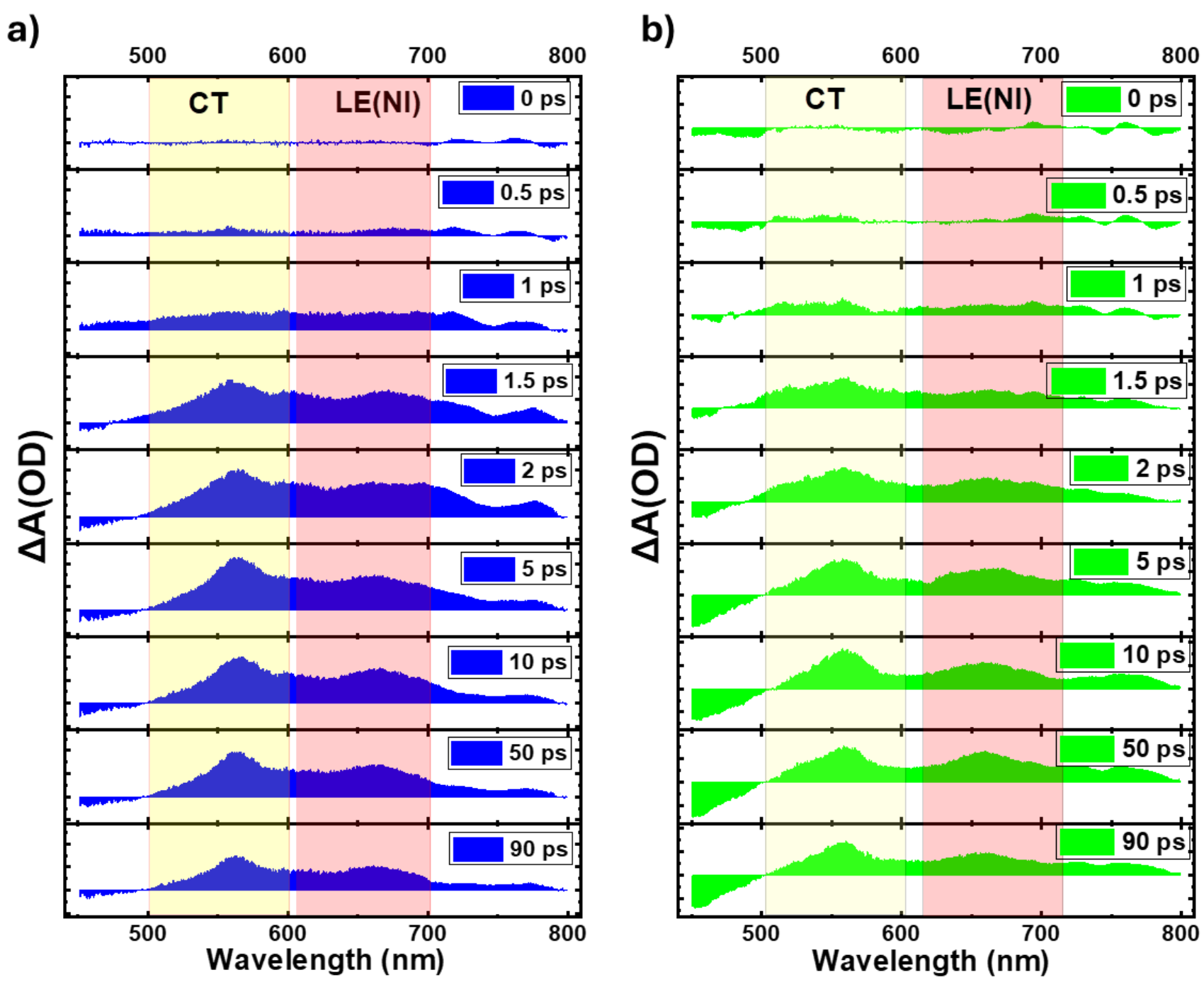

Figure 12S: Transients of A1 and A2 in n-hexane showing ESA bands originating from LE and CT excited states.

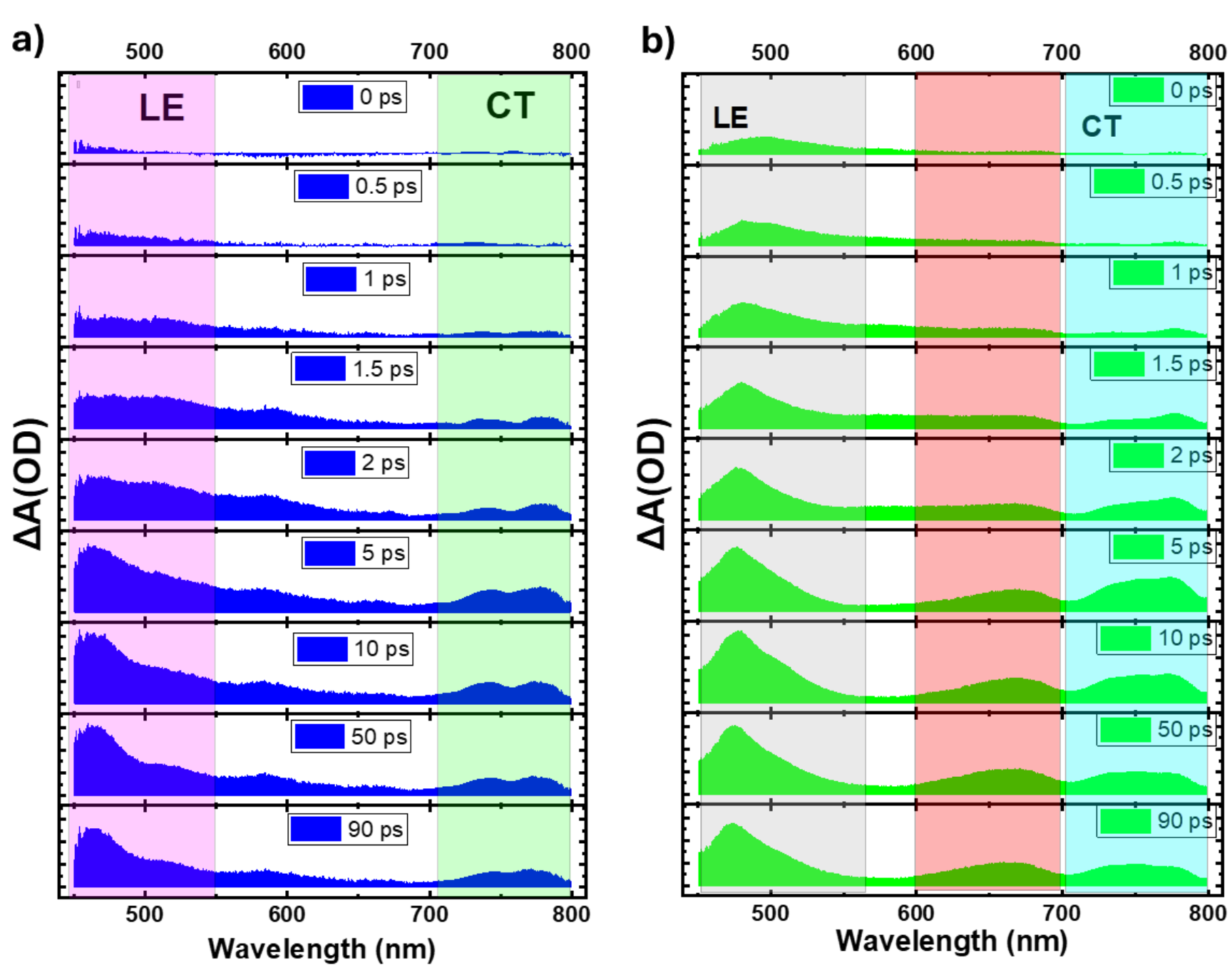

Figure 13S: transient absorptions of A1 and A2 in DCM solvent.

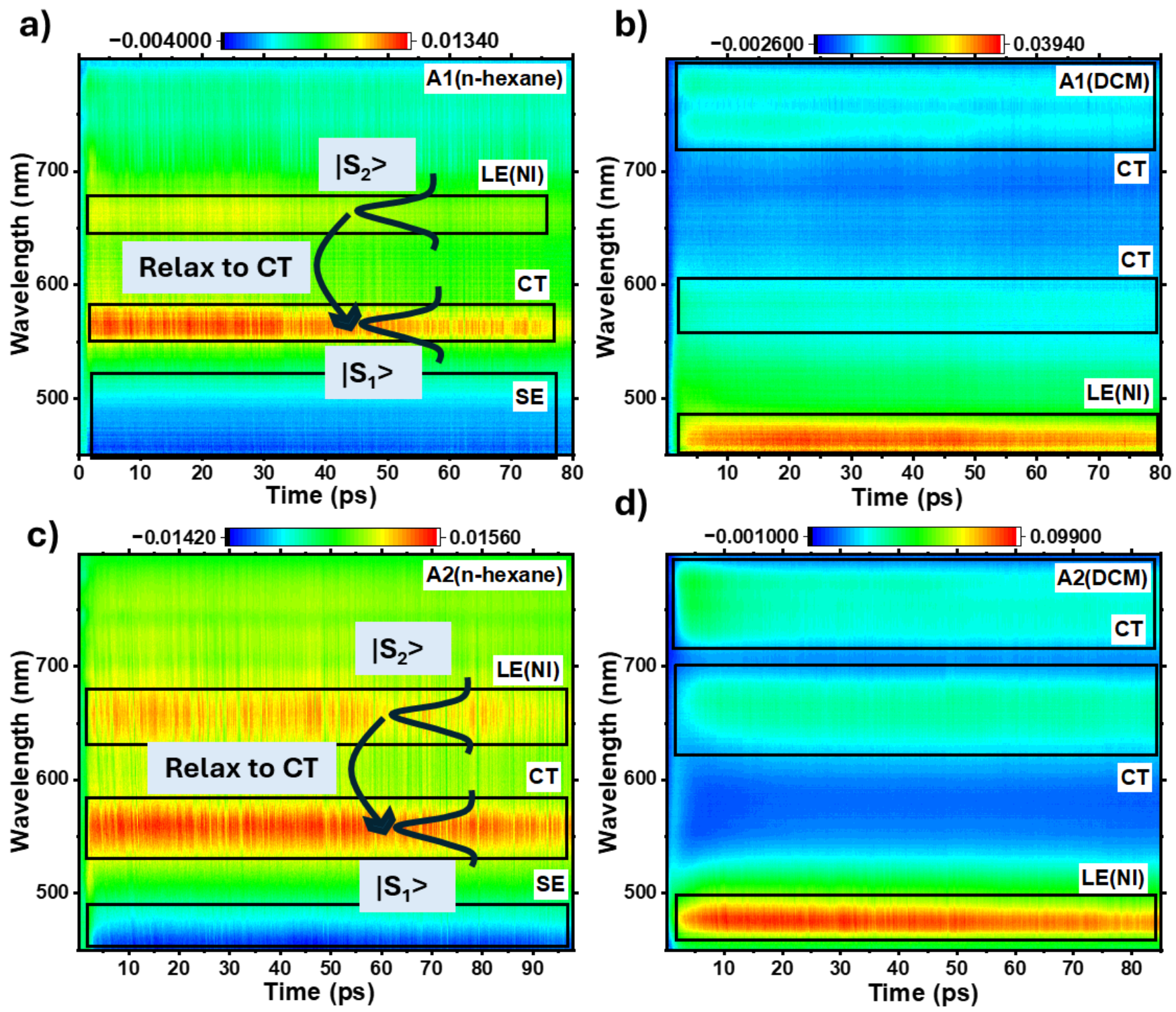

Figure 14S: Transient absorption contours of A1 and A2 in n-hexane **(a and c)** and DCM **(b and d)** up to 90 ps.

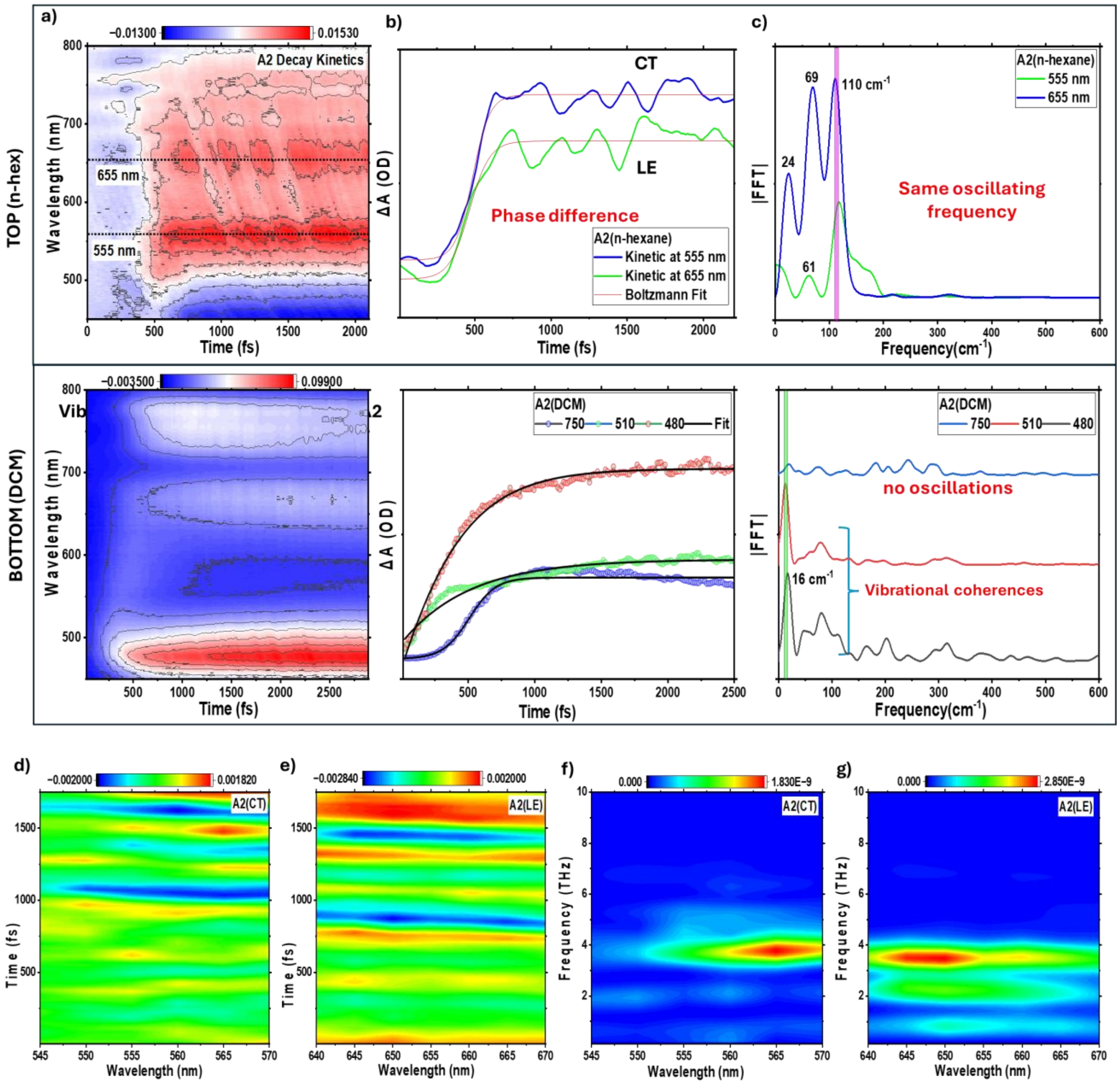

Figure 15S: (a) Decay contours of A2 in the first 2 ps with dotted line showing the probed wavelength used to extract oscillations (**top** shows n-hexane contours), while not obvious in the **bottom** (DCM contours). **(b)** Decay kinetics showing clear modulations superimposed in n-hexane (smoothed to 12 data points), but no such oscillations are observed in DCM. **(c, top)** The FFT power spectrum of the residuals obtained by subtracting the fits from the decay kinetics shows a dominant component in both the CT and LE bands, assigned to vibronic coherences (**TOP**). (**C, bottom**) The CT band shows no oscillatory features, but the LE band shows coherence and is assigned a vibrational nature. **(d-e)** Residual contours show consistent oscillations in the entire LE-CT bands. **(f-g)** FFT power contours of the residuals show that dominant oscillations are consistent in the entire band.

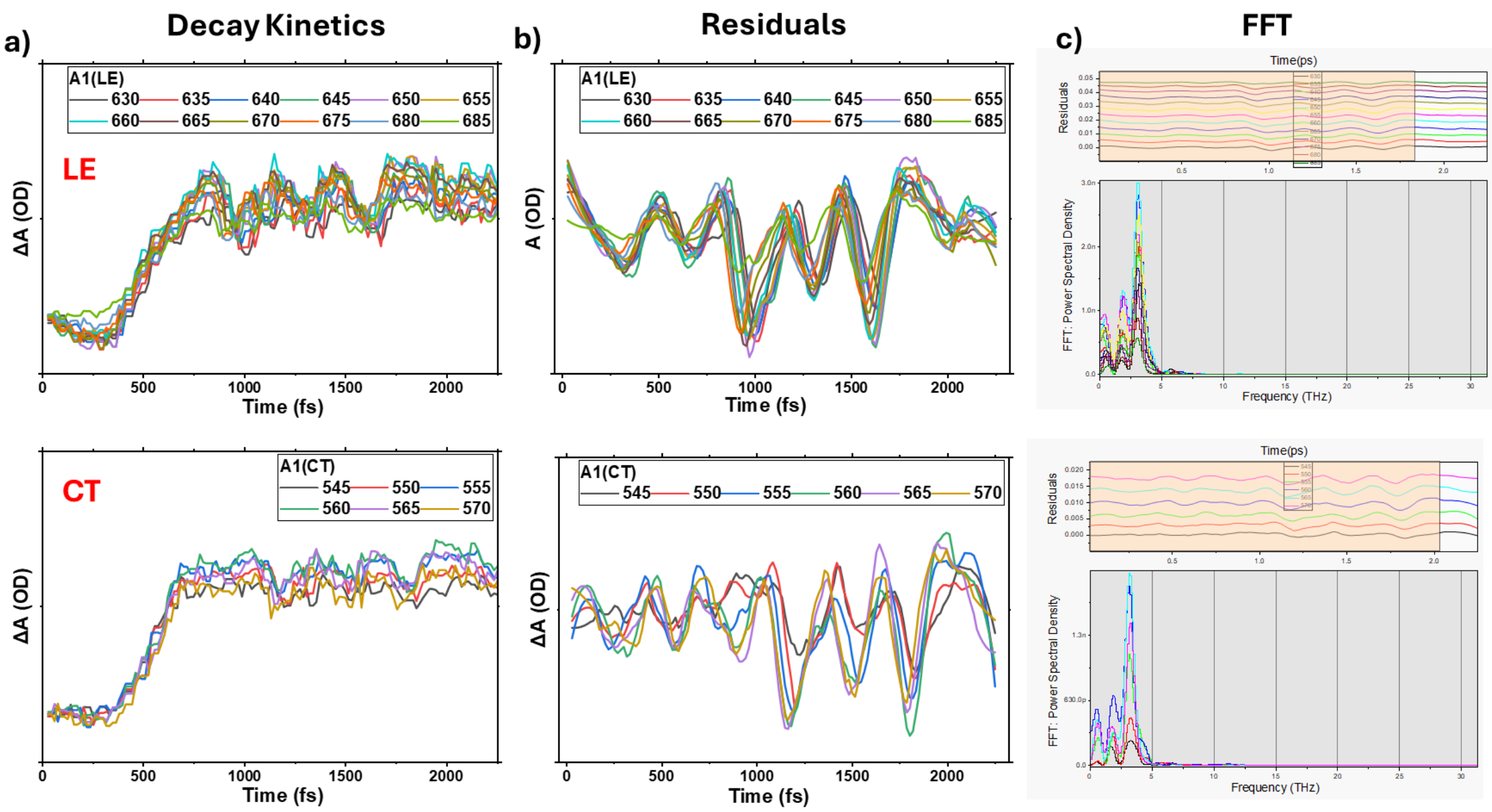

Figure 16S: **(a)** Decay kinetics of A1 in n-hexane at various probe wavelengths of LE and CT bands. **(b)** Residuals of decay fits at the given probe wavelengths (smoothed to 12 data points). **(c)** FFT power spectra of the residuals show the same dominant component at each wavelength.

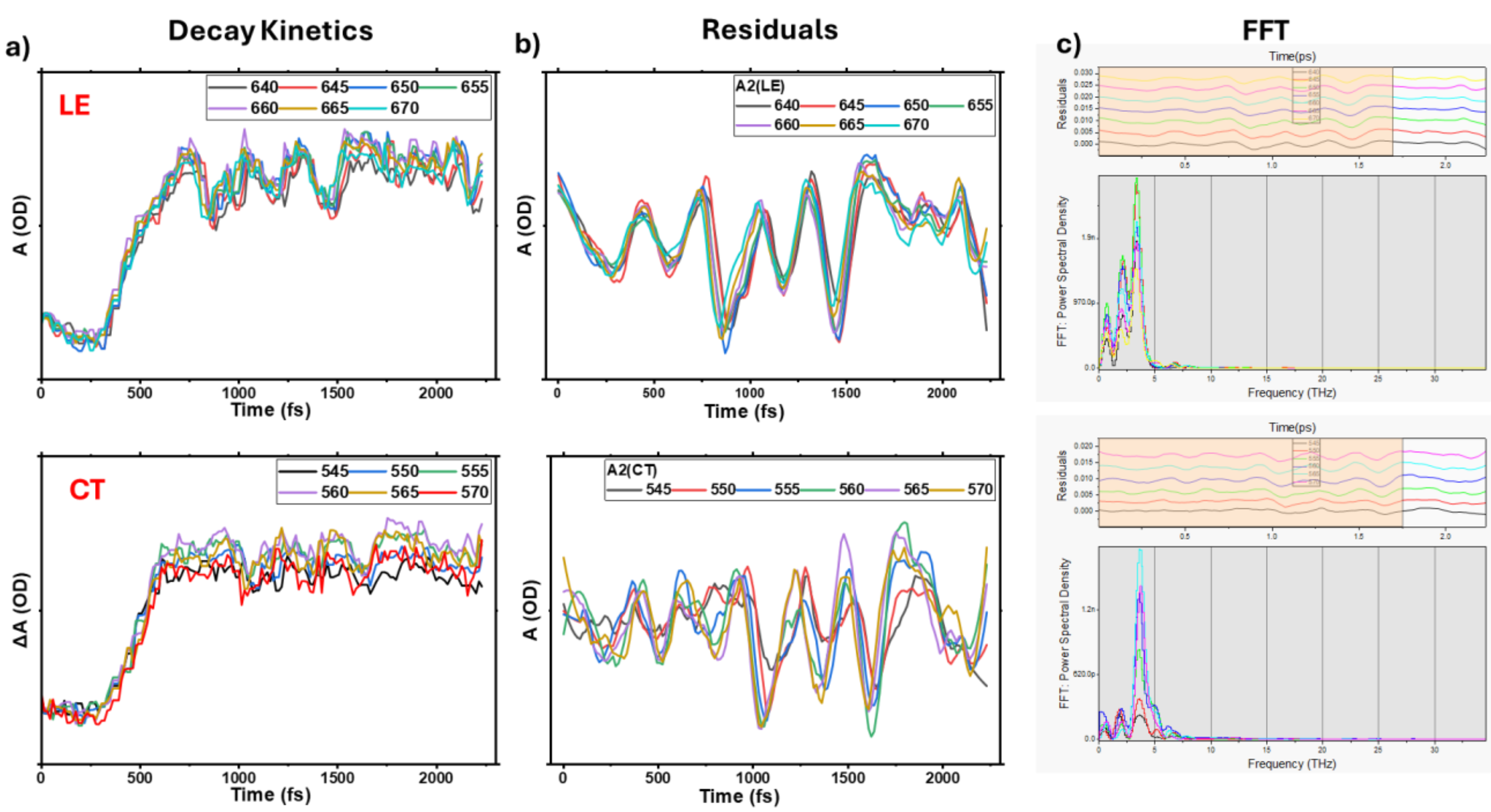

Figure 17S: **(a)** Decay kinetics of A2 in n-hexane at various probe wavelengths of LE and CT bands. **(b)** Residuals of decay fits at the given probe wavelengths (smoothed to 12 data points). **(c)** FFT power spectra of the residuals show the same dominant component at each wavelength.

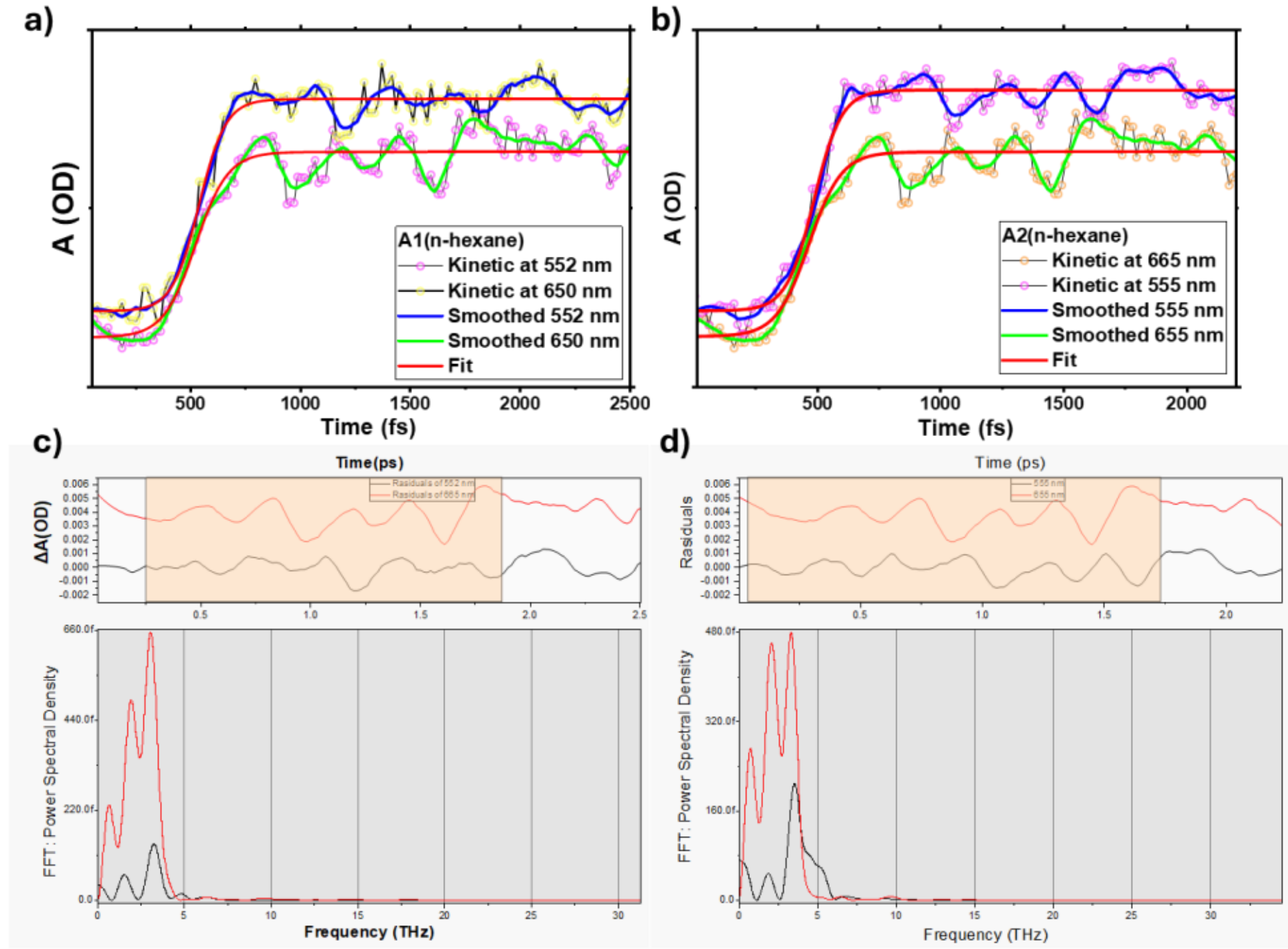

Figure 18S: (a-b) Decay kinetics, smoothed decay kinetics, and their fits. Residuals of kinetics after subtracting the kinetics from the fits of A1 **(c)** and A2 **(d)**. The residuals clearly show modulations in the kinetics. The FFT power spectra of the residuals are also provided.

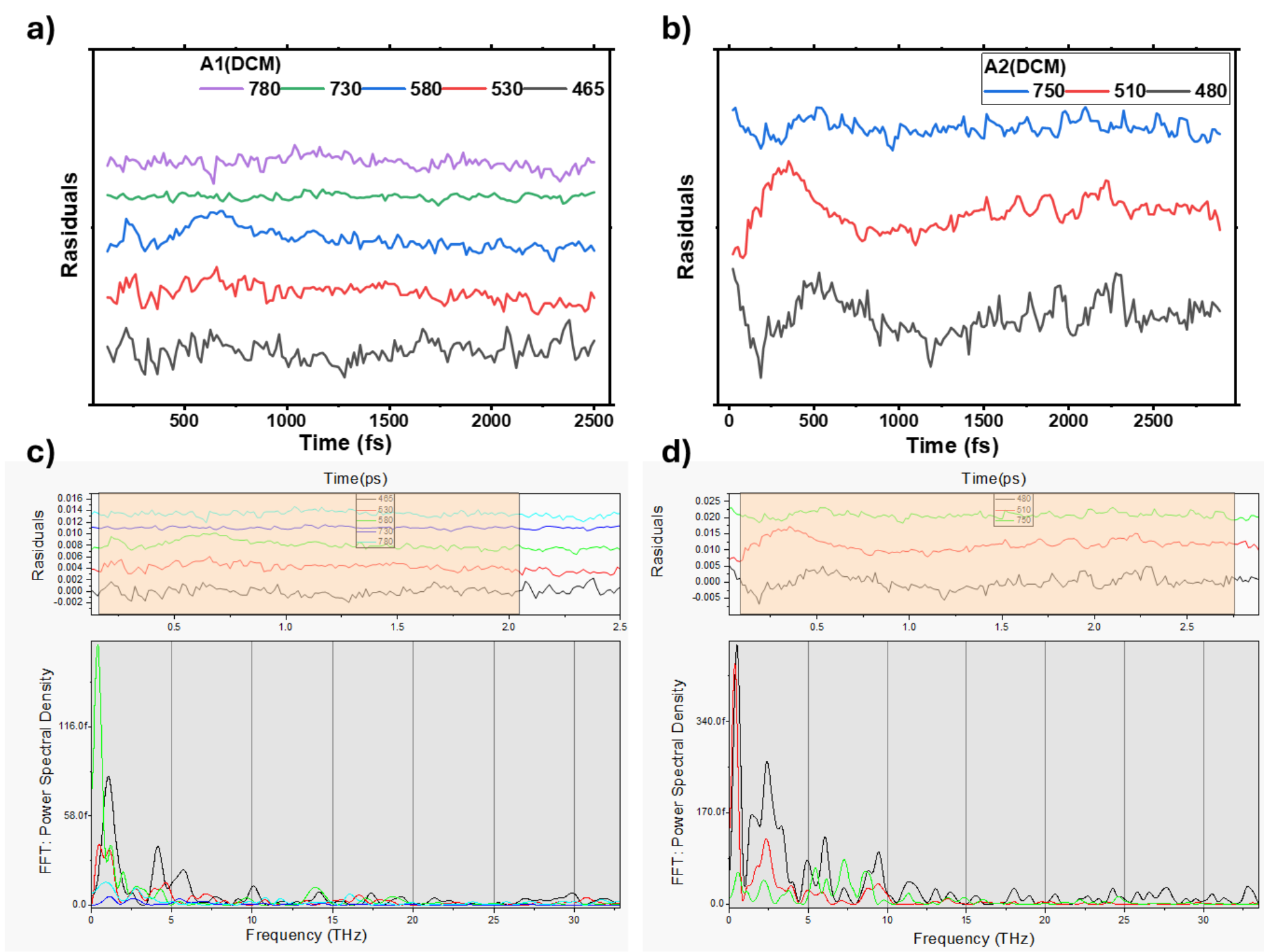

Figure 19S: (a-b) Residuals of kinetics decay in DCM after subtracting fits. Power spectra of the FFT of A1 **(c)** and A2 **(d)**.

A1(CT)

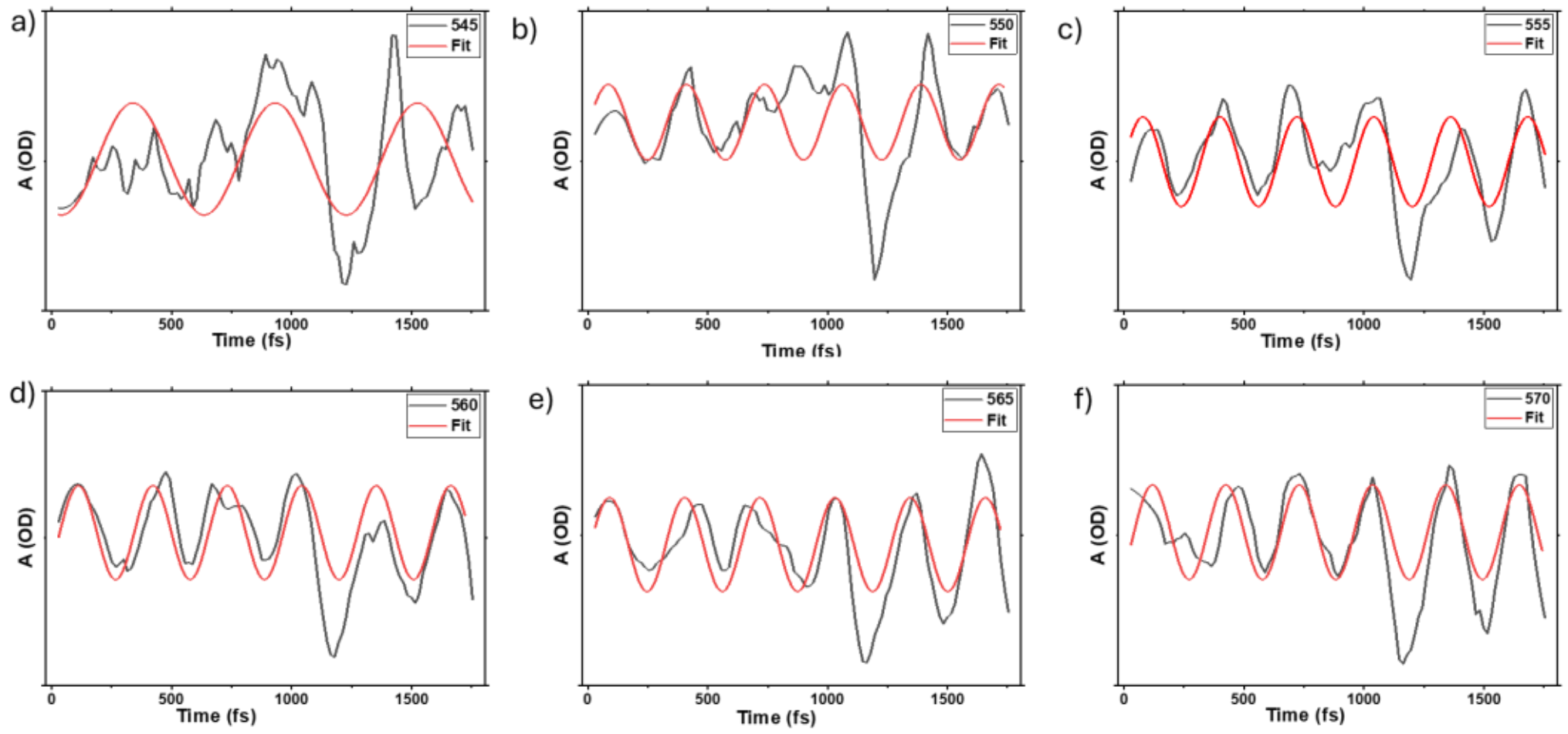
a)
545
Fit
b)
550
Fit
c)
555
Fit
d)
560
Fit
e)
565
Fit
f)
570
Fit
A (OD)
Time (fs)
0
500
1000
1500

A1(LE)

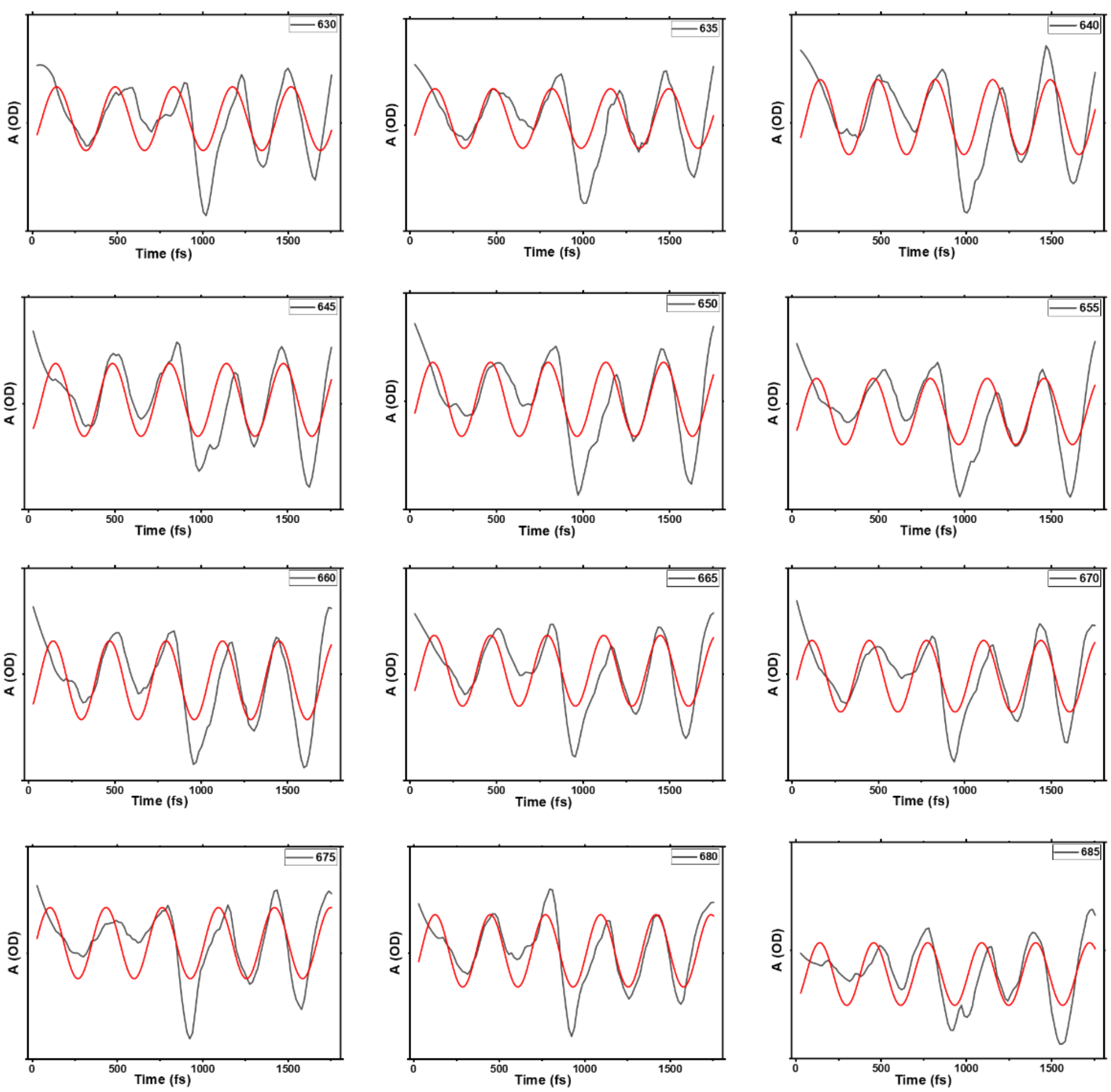
630
635
640
645
650
655
660
665
670
675
680
685
A (OD)
Time (fs)
0
500
1000
1500

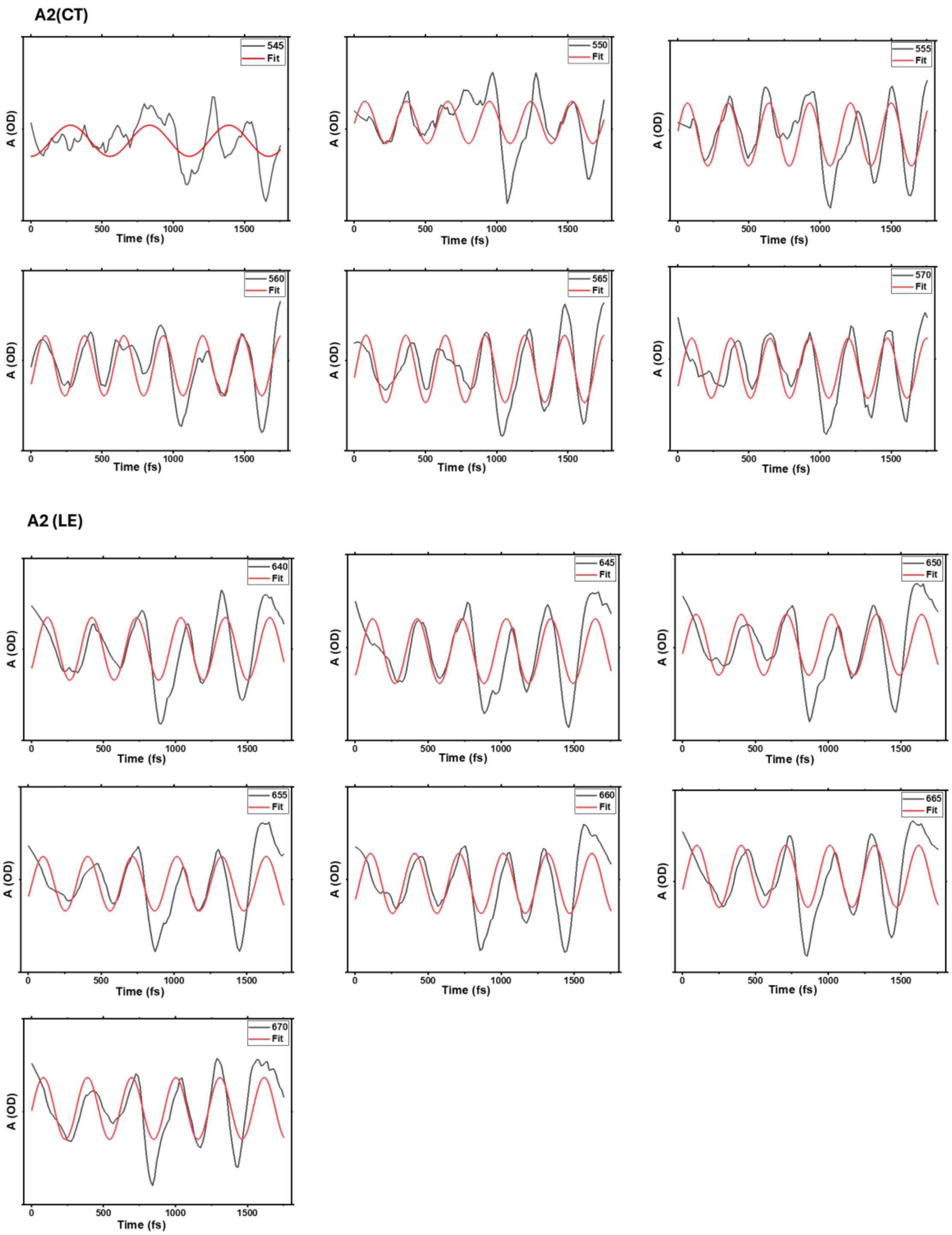

Figure 20S: Sine fits of the residuals of the compound in LE and CT ESA bands.

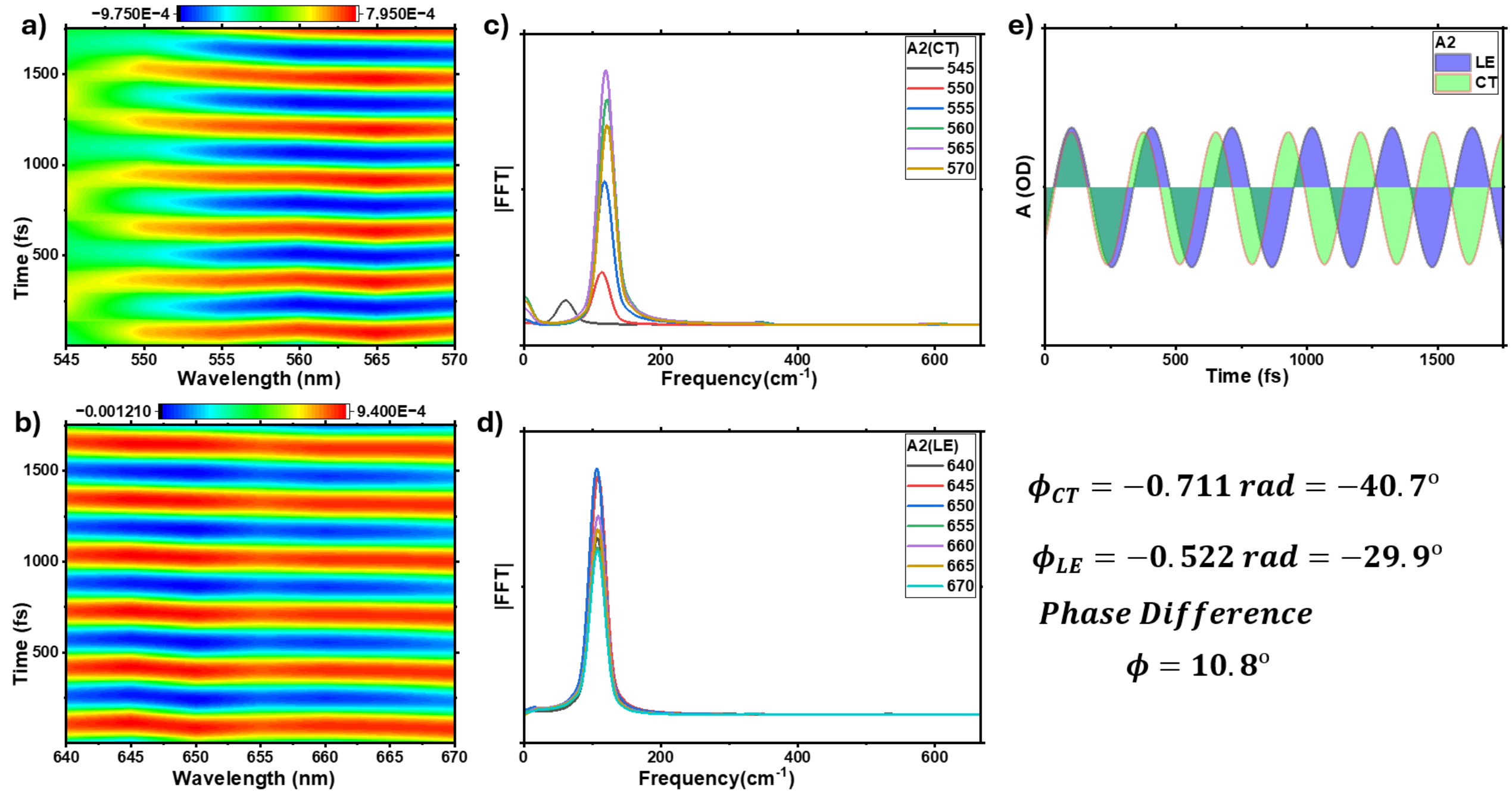

Figure 21S: (a-b) Residuals of decay kinetics after subtracting from fits in n-hexane.

Table 4S: Parameters of sine fits of the residuals at selected wavelengths.

**Model**	**Sine**
**Equation**	**y=y0+A*sin(pi*(x-xc)/w)**
**Plot**	**565 nm (A1_CT)**
**y0**	**-1.53166E-4 ± 3.90018E-20**
**xc**	**0.32463 ± 5.75481E-18**
**w**	**0.1569 ± 1.18335E-18**
**A**	**7.8079E-4 ± 5.18279E-20**

**Model**	**Sine**
**Equation**	**y=y0+A*sin(pi*(x-xc)/w)**
**Plot**	**650 nm (A2_LE)**

y0	-4.36605E-4 ± 4.76526E-20
xc	0.04949 ± 7.00885E-18
w	0.16662 ± 1.17951E-18
A	0.00102 ± 6.53465E-20

Model	Sine
Equation	y=y0+A*sin(pi*(x-xc)/w)
Plot	660 nm (A2_LE)
y0	-1.29773E-4 ± 6.77556E-22
xc	0.02544 ± 1.07752E-19
w	0.15303 ± 1.65158E-20
A	8.78983E-4 ± 9.15139E-22

Model	Sine
Equation	y=y0+A*sin(pi*(x-xc)/w)
Plot	560 nm (A2_CT)
y0	-1.40516E-4 ± 4.40097E-20
xc	0.03129 ± 6.6688E-18
w	0.13823 ± 9.37548E-19
A	8.32166E-4 ± 5.92647E-20

Steady-State Photoluminescence Spectroscopy

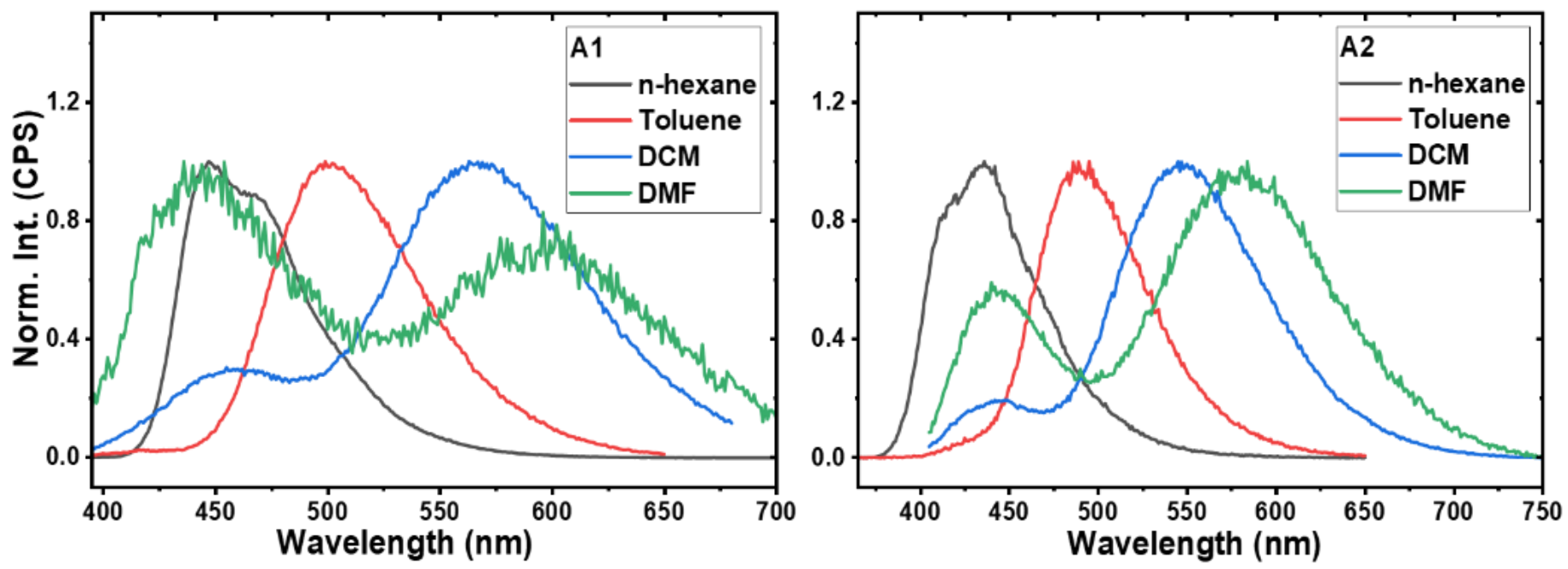

Figure 22S: Solvatochromic emission spectra of the molecules in various solvents showing red shifting of the CT band. In high-polarity solvents, the CT band is redshifted, and an LE band (400-500 nm) emerges.

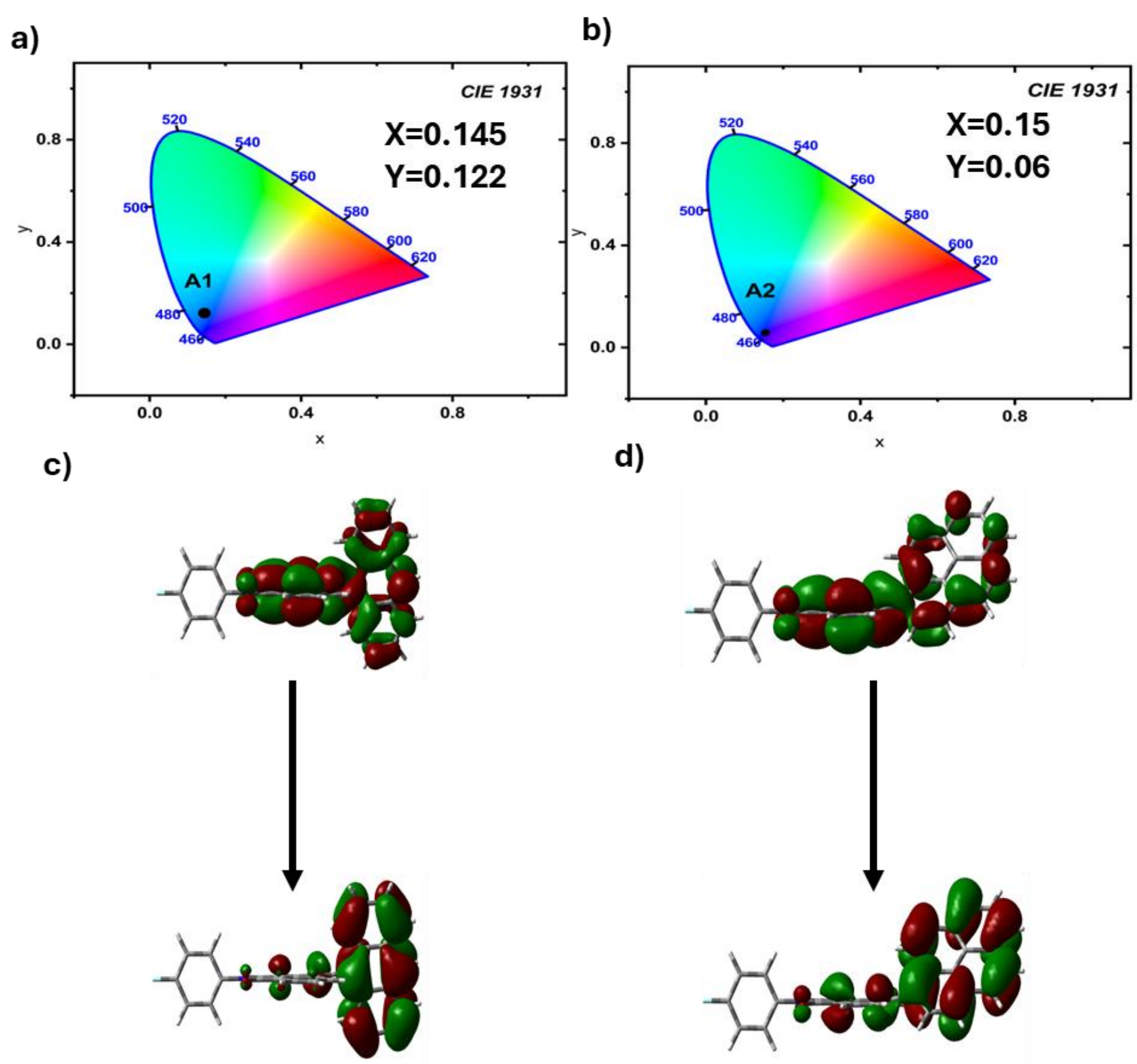

Figure 23S: (a-b) CIE coordinates for A1 (deep blue) and A2 (pure blue) in n-hexane. **(c-d)** The orbitals involved in the emission of both molecules.

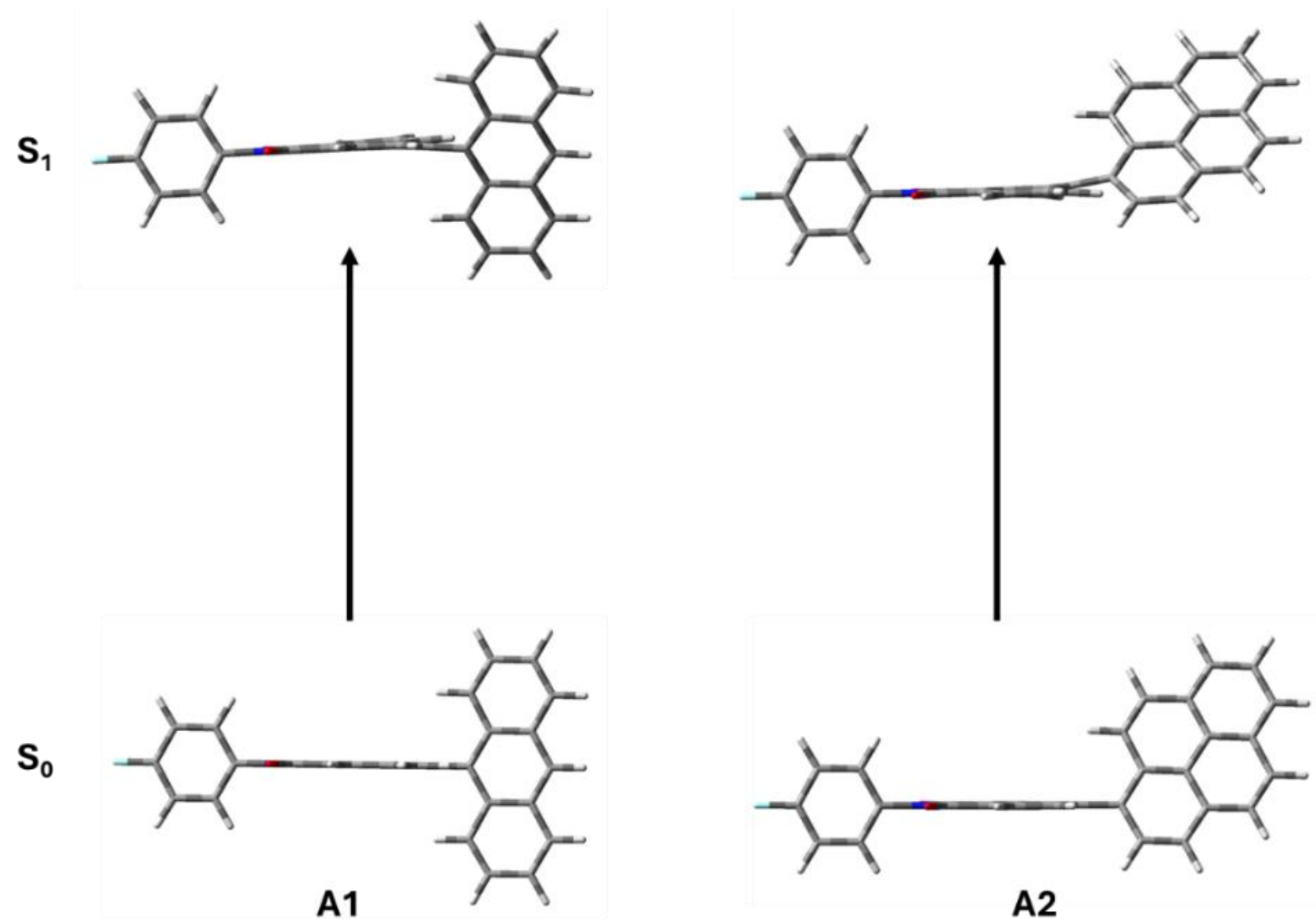

Figure 24S: S_0 and S_1 geometries of the two molecules show extensive planarization.

Table 5S: Dihedral angle between donors (anthracene and pyrene) and the NI ring of the two compounds in the excited state.

Compound	Dihedral angle (donor/NI ring)
**A1**	58.01°
**A2**	43.24°

Table 6S: TDDFT details during emission of the molecules depicting their energies, nature of transition, and respective oscillator strength.

Compound	Energy	Nature of transition	Oscillator strength (f)
**A1**	2.81	CT	0.3204
**A2**	2.94	CT	0.7708

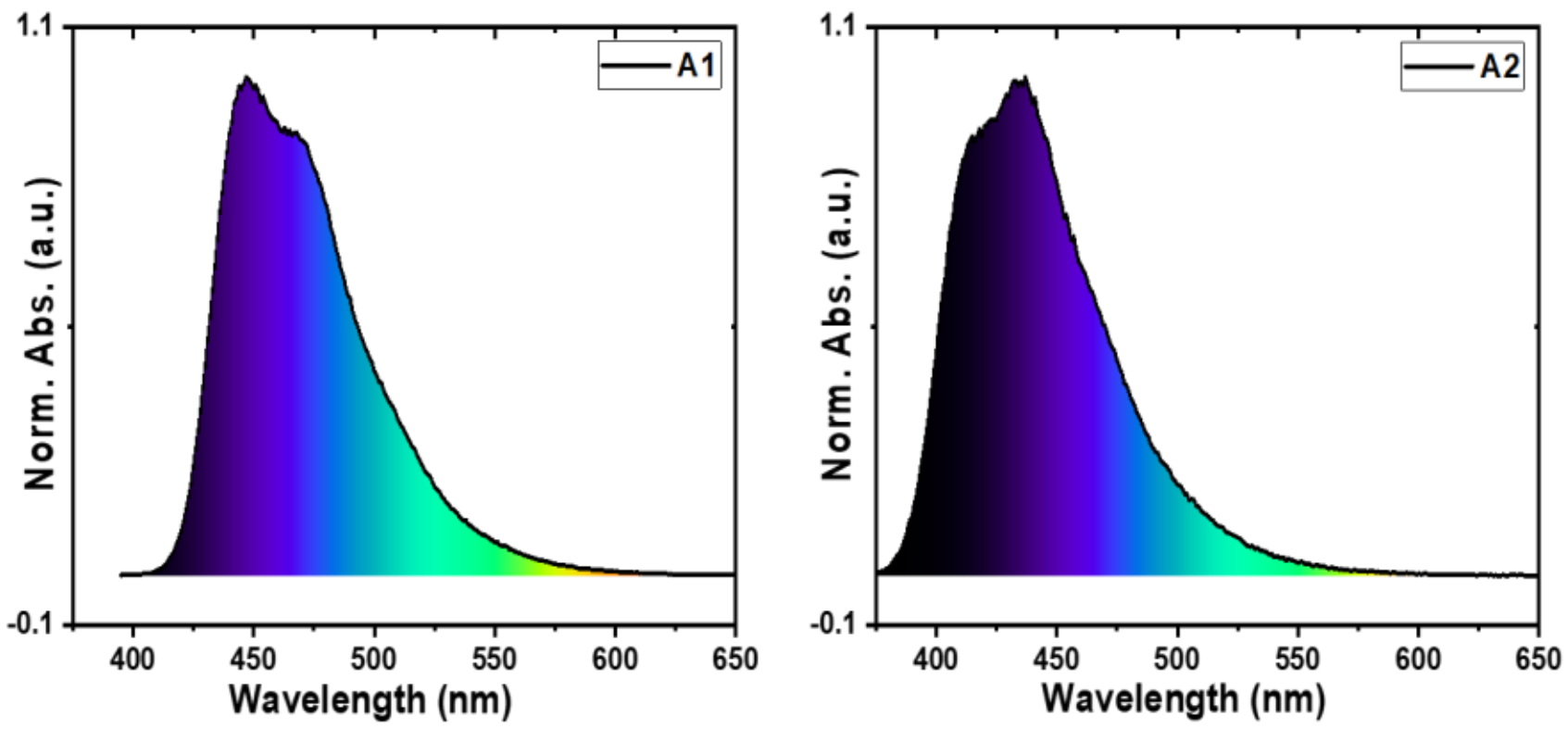

Figure 25S: Color spectra of the emission in n-hexane.

Table 7S: CIE 1931 coordinates of the molecules compared with pure blue coordinates. Full width at half maxima (FWHM) of the compounds are presented.

Compound	CIE_X	CIE_Y	FWHM
**A1**	0.145	0.122	61.4
**A2**	0.15	0.06	68.9
**Pure Blue**	0.15	0.06	-

Time-Gated Photoluminescence Spectroscopy (TGPL)

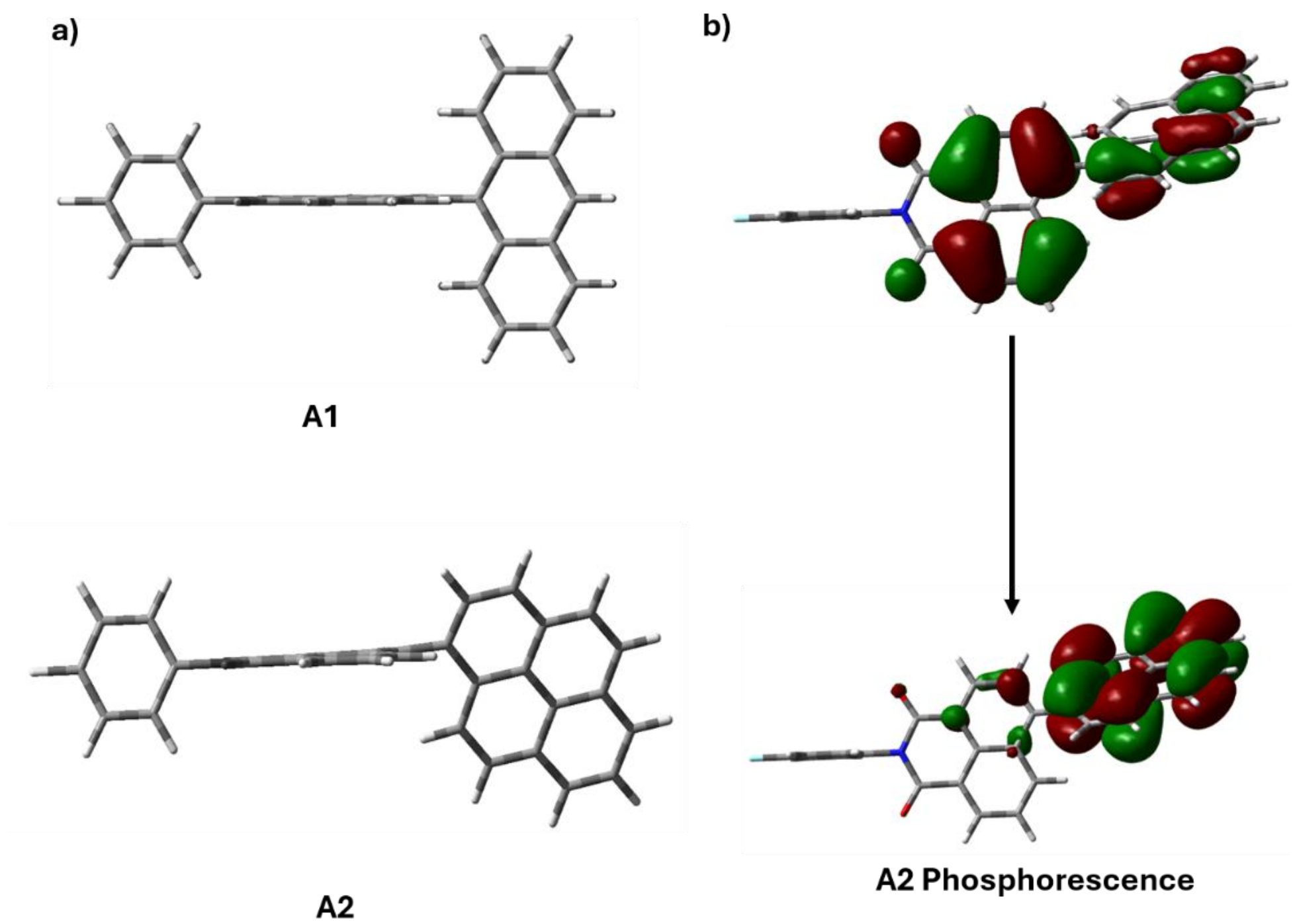

Figure 26S: (a) Triplet geometry of the molecules. (b) Particle-hole (electron-hole) representation of the $T_1 \rightarrow S_0$ phosphorescence transition of A2, showing charge redistribution from the NI-centered orbital (HOMO) toward the pyrene, consistent with predominant ^{3}CT character of the triplet state.

Table 8S: Spin-orbit coupling (in cm^{-1}) between singlet and closely situated triplet manifolds.

Compound	SOC (cm^{-1})
**NI**	23.6
**A1**	1.06
**A2**	4.14

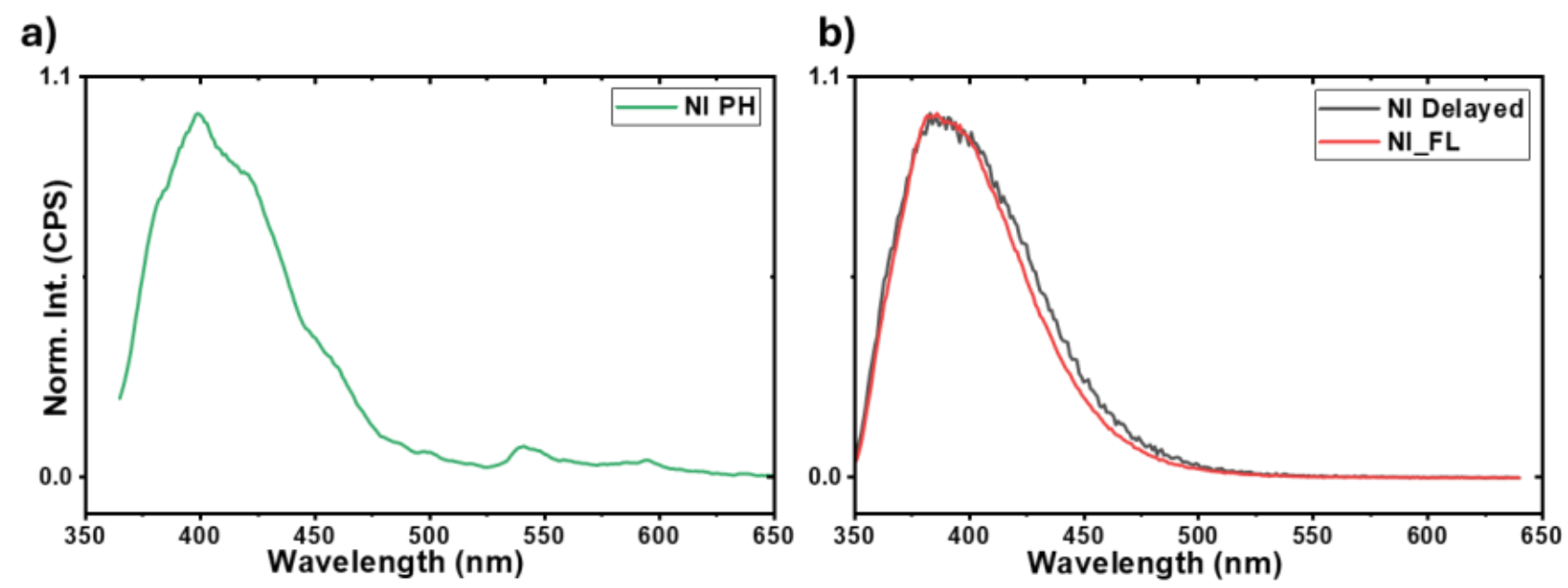

Figure 27S: (a) TGPL spectra of the NI acceptor ring with a 50 µs delay at 77K in toluene. **(b)** TGPL spectra of the NI ring compared with its SSPL spectra at RT. The spectra show TTA delayed fluorescence.

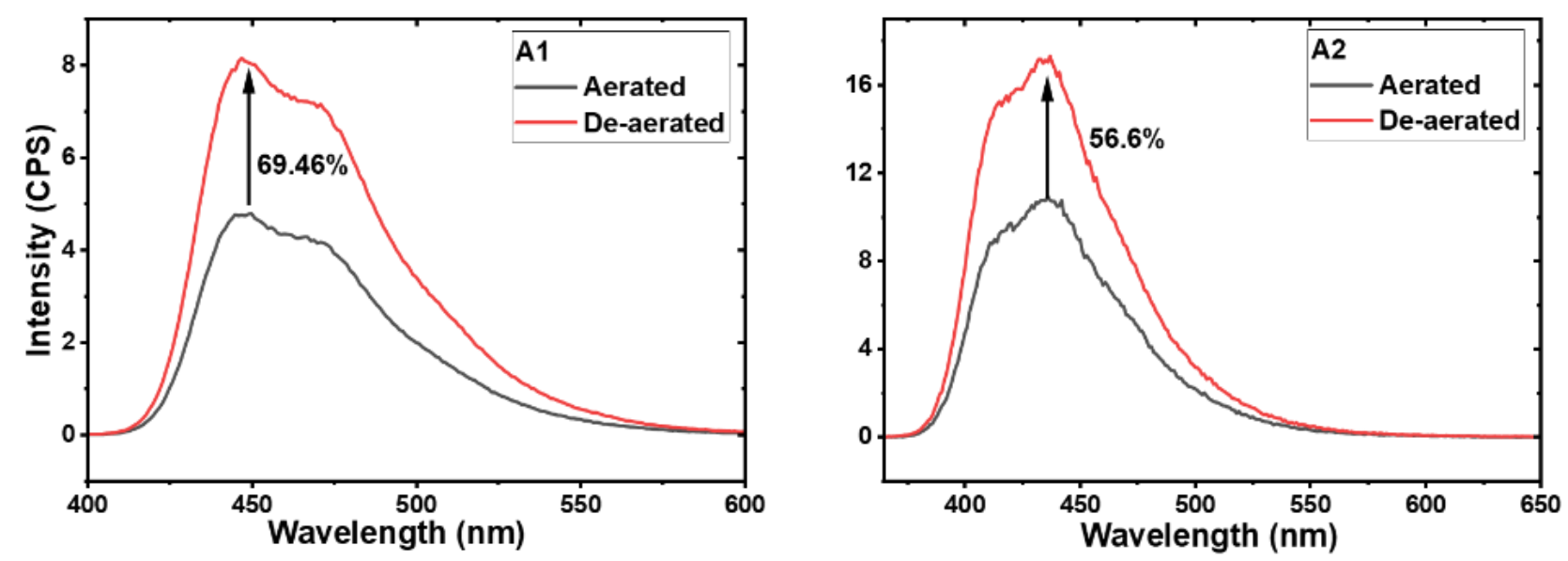

Figure 28S: Aerated and de-aerated experiments in n-hexane for A1 and A2 molecules. Aerated represents the solvent taken directly without any nitrogen/oxygen purging, while de-aerated represents a nitrogen-purged solvent.

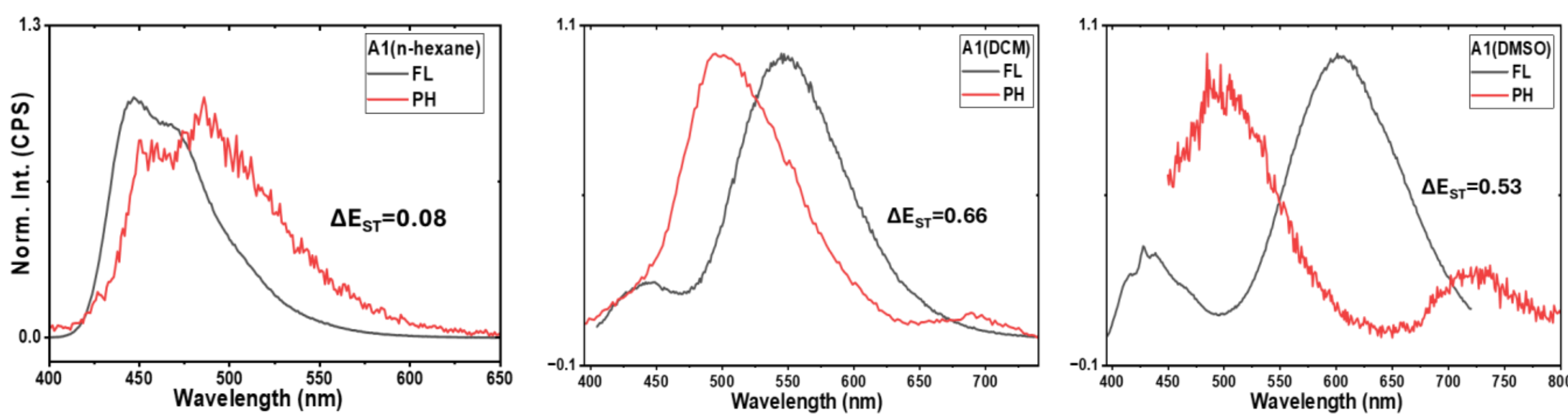

Figure 29S: Steady-state and time-gated emission in various solvents. Time-gate spectra were acquired with a 50 µs delay at 77K.

References

[1] M. J. Frisch, G. W. Trucks, H. B. Schlegel, G. E. Scuseria, M. A. Robb, J. R. Cheeseman, G. Scalmani, V. Barone, G. A. Petersson, H. Nakatsuji, X. Li, M. Caricato,

A. V. Marenich, J. Bloino, B. G. Janesko, R. Gomperts, B. Mennucci, H. P. Hratchian, J. V. Ortiz, A. F. Izmaylov, J. L. Sonnenberg, Williams, F. Ding, F. Lipparini, F. Egidi, J. Goings, B. Peng, A. Petrone, T. Henderson, D. Ranasinghe, V. G. Zakrzewski, J. Gao, N. Rega, G. Zheng, W. Liang, M. Hada, M. Ehara, K. Toyota, R. Fukuda, J. Hasegawa, M. Ishida, T. Nakajima, Y. Honda, O. Kitao, H. Nakai, T. Vreven, K. Throssell, J. A. Montgomery Jr., J. E. Peralta, F. Ogliaro, M. J. Bearpark, J. J. Heyd, E. N. Brothers, K. N. Kudin, V. N. Staroverov, T. A. Keith, R. Kobayashi, J. Normand, K. Raghavachari, A. P. Rendell, J. C. Burant, S. S. Iyengar, J. Tomasi, M. Cossi, J. M. Millam, M. Klene, C. Adamo, R. Cammi, J. W. Ochterski, R. L. Martin, K. Morokuma, O. Farkas, J. B. Foresman, D. J. Fox, Wallingford, CT, **2016**.

[2] aR. Krishnan, J. S. Binkley, R. Seeger, J. A. Pople, J. Chem. Phys. **1980**, 72, 650-654; bV. A. Rassolov, J. A. Pople, M. A. Ratner, T. L. Windus, J. Chem. Phys. **1998**, 109, 1223-1229; cJ. M. Galbraith, A. M. James, C. T. Nemes, Mol. Phys. **2014**, 112, 654-660; dZ. Rashid, J. H. van Lenthe, J. Chem. Phys. **2013**, 138.

[3] aT. Kobayashi, Z. Wang, T. Otsubo, J. Phys. Chem. A **2007**, 111, 12985-12994; bY.-C. Wei, L.-Y. Hsu, J. Phys. Chem. Let. **2023**, 14, 2395-2401.
